# A succinylation‐based classifier predicts chemotherapy response in prostate cancer and reveals KAT2A as a therapeutic target

**DOI:** 10.1002/ctm2.70737

**Published:** 2026-07-07

**Authors:** Antao Dong, Wei Hu, Zhihui Lu, Liangliang Li, Kun Liu, Shiyao Feng, Dongdong Xie

**Affiliations:** ^1^ Department of Urology The Fifth Affiliated Hospital of Anhui Medical University Fuyang Anhui Province China; ^2^ Department of Urology The Second Affiliated Hospital of Anhui Medical University Hefei Anhui Province China; ^3^ Department of Urology The Fourth Affiliated Hospital of Anhui Medical University Hefei Anhui Province China

**Keywords:** chemoresistance, KAT2A, prostate cancer, succinylation, tumour microenvironment

## Abstract

**Background:**

Chemotherapy resistance remains a critical hurdle in advanced prostate cancer (PCa). Succinylation, an essential post‐translational modification linking cellular metabolism with epigenetic regulation, has been implicated in tumour progression; however, its contribution to PCa chemoresistance remains poorly defined.

**Objective:**

This study aimed to evaluate the prognostic significance of succinylation in PCa, develop a succinylation‐based biomarker, and elucidate the mechanisms driving chemotherapy resistance.

**Methods:**

We generated a succinylation score (SS) by applying single‐sample gene set enrichment analysis (ssGSEA) to transcriptomic profiles from the TCGA‐PRAD cohort. Its relationships with survival, the tumour microenvironment (TME), and treatment susceptibility were examined using CIBERSORT, GSEA, TIDE, oncoPredict, and single‐cell RNA sequencing (scRNA‐seq). Findings were functionally validated in patient‐derived organoids, PCa cell lines, and xenograft models through genetic manipulation, chemosensitivity assays, and mechanistic studies.

**Results:**

High SS correlated with favourable prognosis, lower Gleason scores, absent lymph node metastasis, and an immune‐active TME enriched in CD8^+^ precursor exhausted T cells. High‐SS tumours showed enhanced sensitivity to docetaxel and cisplatin. scRNA‑seq identified KAT2A as a key driver in low‑SS malignant clusters with chemoresistance features. KAT2A was elevated in chemoresistant tissues, cell lines, and organoids. KAT2A knockout sensitised cells to chemotherapy, while ectopic expression promoted resistance in vitro and in vivo. Mechanistically, KAT2A‐mediated succinylation of PIK3R2 at K477 and K564 inhibited its ubiquitination and proteasomal degradation, stabilising PIK3R2 to drive chemoresistance. The KAT2A inhibitor Butyrolactone 3 synergised with standard chemotherapy to suppress tumour growth.

**Conclusion:**

The succinylation score serves as a robust prognostic biomarker integrating metabolic and immunological features in PCa. The KAT2A‐PIK3R2 succinylation pathway represents a newly defined driver of chemoresistance and points to MB‐3‐based combination therapy as a potential strategy for resistant advanced disease.

**Key points:**

- A transcriptome‐based succinylation score (SS) stratifies prostate cancer by prognosis, immune contexture, and chemotherapy sensitivity.
- KAT2A is enriched in low‐SS, chemoresistant tumours and promotes resistance by succinylating PIK3R2.
- KAT2A‐mediated succinylation competitively inhibits PIK3R2 ubiquitination, stabilising PIK3R2 and driving chemoresistance.
- The KAT2A inhibitor MB‐3 synergises with chemotherapy to suppress tumour growth in both chemoresistant and chemosensitive models.

## Graphical abstract

1. A transcriptome‐based succinylation score (SS) stratifies prostate cancer by prognosis, immune contexture, and chemotherapy sensitivity.
2. KAT2A is enriched in low‐SS, chemoresistant tumours and promotes resistance by succinylating PIK3R2.
3. KAT2A‐mediated succinylation competitively inhibits PIK3R2 ubiquitination, stabilising PIK3R2 and driving chemoresistance.
4. The KAT2A inhibitor MB‐3 synergises with chemotherapy to suppress tumour growth in both chemoresistant and chemosensitive models.

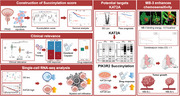

## 1 INTRODUCTION

Prostate cancer is one of the most frequently diagnosed cancers in men and remains a major cause of cancer mortality worldwide.^1^ Although surgery, radiotherapy, and androgen‐deprivation therapy are effective for many patients with localised disease, treatment options become far more limited after resistance develops in advanced tumours.^2^, ^3^ Chemotherapy, particularly docetaxel‐based regimens, continues to play an important role in selected patients with advanced disease. However, both intrinsic and acquired chemoresistance substantially compromise treatment efficacy and are closely associated with disease progression and poor survival.^4^ Hence, there is an urgent need for biologically grounded biomarkers that can refine risk assessment and forecast treatment response in advanced prostate cancer.

Accumulating evidence points to post‐translational modifications (PTMs) as pivotal regulators of tumour evolution, therapeutic adaptation, and signalling plasticity.^5^ Among these modifications, lysine succinylation has emerged as a metabolically sensitive regulatory mechanism capable of modulating protein structure, enzymatic activity, subcellular localisation, and transcriptional programs.^6^, ^7^ Because succinylation is tightly coupled to succinyl‐CoA availability and mitochondrial metabolism, it provides a potential interface between metabolic rewiring and malignant progression.^8^ Recent studies have implicated aberrant succinylation in several cancer‐related processes, including tumour growth, metastasis, and therapeutic resistance.^9^, ^10^ Even so, its biological and clinical relevance in prostate cancer, especially with regard to chemotherapy response, remains incompletely characterised.

This question is particularly relevant because the tumour metabolic state has broad consequences beyond cancer cell‐intrinsic survival. Metabolic reprogramming can shape the tumour microenvironment (TME), influence immune‐cell recruitment and activation, and alter the balance between immune stimulation and immune suppression.^11^, ^12^ At the same time, metabolic adaptation enables tumour cells to withstand cytotoxic stress, thereby contributing to chemotherapy tolerance.^13^ Given that succinylation‐related genes are closely linked to metabolic programs, and that these programs influence both malignant‐cell fitness and immune contexture, we reasoned that a transcriptome‐derived succinylation‐related signature could capture an integrated tumour state relevant to immune status and chemosensitivity.^14^ Within this framework, a global succinylation score would not merely reflect a single isolated biochemical event, but rather a systems‐level state linking metabolism, tumour ecology, and treatment response.

Despite this rationale, no study has systematically defined succinylation‐related tumour states in prostate cancer or examined their relationship with prognosis, immune contexture, and chemotherapy sensitivity.^15^ Moreover, the tumour‐intrinsic effectors that may drive chemoresistance within distinct succinylation‐defined states remain largely unknown.^16^, ^17^ Addressing these questions is important not only for advancing the mechanistic understanding of prostate cancer progression, but also for identifying actionable therapeutic targets capable of overcoming drug resistance.

In the present study, we established a transcriptome‐based succinylation score (SS) to characterise succinylation‐related states in prostate cancer and to evaluate their clinical, immunological, and therapeutic relevance. We found that SS stratified tumours into biologically distinct states associated with prognosis, immune landscape, and predicted chemotherapy response. Integrative analyses further identified KAT2A as a resistance‐associated effector enriched in low‐SS tumours. Functional studies in patient‐derived organoids, prostate cancer cell models, and xenografts demonstrated that KAT2A promotes chemoresistance by stabilising PIK3R2 through suppressing its ubiquitination in a succinylation‐dependent manner. Collectively, our findings establish a conceptual and mechanistic link between succinylation‐related tumour states and chemotherapy response, and identify the KAT2A‐PIK3R2 succinylation axis as a candidate therapeutic target in advanced prostate cancer.

## 2 MATERIALS AND METHODS

### 2.1 Data acquisition and bioinformatics analysis

RNA sequencing data and matched clinical information for prostate adenocarcinoma (PRAD) were obtained from The Cancer Genome Atlas (TCGA) through the UCSC Xena platform. Samples lacking survival information or with survival times under 30 days were excluded to minimise early censoring bias. scRNA‐seq data were downloaded from the Gene Expression Omnibus (GEO) database under accession number GSE137829, including gene expression matrices and cell‐type annotation information.^18^ A set of succinylation‐related genes was compiled from the GeneCards database using a relevance score threshold > 5 to capture genes with robust database‐supported associations with succinylation biology. This gene set served as a transcriptomic proxy for succinylation‐related pathways rather than a comprehensive list of experimentally validated succinylated proteins or residues.

For scRNA‐seq analysis, Seurat (version 4.1.0) was applied for data processing. Cells were kept if they met the following criteria: more than 500 detected genes, over 1000 UMIs, and less than 15% mitochondrial transcripts.^19^, ^20^ Data normalisation was performed using the LogNormalize method, after which the top 2000 variable genes were chosen for downstream analysis. Principal component analysis (PCA) was applied, and Harmony was used to correct batch effects.^21^ The first 20 Harmony‐adjusted principal components were applied for uniform manifold approximation and projection (UMAP) and construction of the k‐nearest neighbour graph. Cell clustering was then carried out with a resolution parameter of 0.5. Major cell populations were identified according to canonical marker genes, including B cells (CD19, CD79A), endothelial cells (VWF, PECAM1, EMCN), epithelial cells (EPCAM, KRT18, KRT19), fibroblasts (COL1A1, ACTA2, PDGFRA), myeloid cells (CD68, CD163, FCN1), and T/NK cells (CD3D, CD8A, IL7R).

### 2.2 Transcriptome profiling and functional analysis

A succinylation score (SS) for each TCGA‐PRAD sample was derived using single‐sample gene set enrichment analysis (ssGSEA) in the GSVA package based on a predefined succinylation‐associated gene set. Because ssGSEA relies on gene ranking rather than weighted expression values, no individual gene weights were applied. Therefore, the SS was considered a transcriptome‐based surrogate reflecting the succinylation‐related tumour state, rather than a direct indicator of overall protein succinylation levels. Patients were subsequently classified into high‐ and low‐SS groups using the median SS as the cutoff.

For the scRNA‐seq dataset, patient‐level pseudobulk expression matrices were generated by aggregating gene expression profiles across cells from the same patient. The SS of each patient was then computed using the identical gene set and ssGSEA method. Based on the median SS, patients were further categorised into high‐ and low‐SS groups, and all cells from each individual were assigned the corresponding patient‐level SS status for downstream analyses. This strategy allowed the bulk‐defined succinylation state to be projected onto single‐cell populations while maintaining the biological characteristics of each patient.

Differential expression analysis between tumour and normal samples, as well as between high‐SS and low‐SS groups, was performed using limma (v3.50.0).^22^ Raw count matrices were transformed with voom for variance stabilisation, followed by linear fitting and empirical Bayes moderation. Genes with |log2 fold change| greater than 1 and FDR below 0.05 were regarded as significant. Gene Set Enrichment Analysis (GSEA) was subsequently conducted with clusterProfiler against Reactome gene sets using 1000 permutations.

### 2.3 Clinical correlation and therapeutic response prediction

Associations between gene expression and biochemical recurrence were examined with univariate Cox proportional hazards models. Survival differences were assessed by Kaplan–Meier curves with log‐rank testing. Independent prognostic value was evaluated using multivariable Cox regression models incorporating age, N stage, and Gleason score.

Immune infiltration levels were inferred with CIBERSORT, and Spearman correlation analysis was conducted to determine the relationships between SS and immune‐cell fractions. The likelihood of response to immune checkpoint blockade was subsequently estimated using the TIDE framework.^23^ Predicted sensitivity to chemotherapeutic agents was derived with the oncoPredict R package trained on the GDSC2 pharmacogenomics dataset,^24^ and Wilcoxon rank‐sum testing was performed to evaluate differences in the predicted IC50 values of cisplatin and docetaxel between SS‐defined subgroups.

Because CIBERSORT, TIDE, and oncoPredict are transcriptome‐based inference frameworks, their outputs may be influenced by sample‐level characteristics such as tumour purity and stromal composition. In the present study, all samples were analysed within the uniformly processed TCGA‐PRAD cohort using a consistent normalisation pipeline, which helped reduce technical variability. These results were therefore interpreted as relative sample‐level associations rather than direct measures of absolute immune abundance or real‐world clinical drug response.

### 2.4 Immunohistochemistry

Paired prostate tumour tissues and adjacent histologically non‐tumourous samples were collected from patients at The Second Hospital of Anhui Medical University. Ethical approval for the use of human specimens was granted by the hospital's ethics committee (Approval No. SL‐YX2023‐167), and all enrolled individuals provided written informed consent in advance.

Immunohistochemical staining was performed on 10 pairs of formalin‐fixed, paraffin‐embedded (FFPE) prostate specimens. Briefly, 4‐µm sections were dewaxed, rehydrated, and subjected to citrate‐based antigen retrieval, followed by endogenous peroxidase blocking with 3% H_2_O_2_. KAT2A/GCN5 expression was detected using a commercial IHC kit (Proteintech) according to the manufacturer's protocol. Microscopic images were acquired with a Nikon Eclipse upright microscope, and staining signals were evaluated independently by two board‐certified pathologists using ImageJ for quantification.

### 2.5 Patient‐derived organoid culture and characterisation

Tumour tissues freshly obtained from patients with prostate cancer were dissociated in DMEM/F12 containing collagenase II (1 mg/mL; Gibco) together with 10 µM Y‐27632 (TargetMol). The resulting cell suspension was passed through a filter, resuspended in Matrigel (Corning), and plated as 50 µL domes. After gel solidification, the domes were maintained in complete organoid medium prepared with advanced DMEM/F12 and supplemented with B27 (1×; Gibco), N‐acetylcysteine (1.25 mM), EGF (50 ng/mL), Noggin (100 ng/mL), R‐spondin 1 (100 ng/mL), DHT (10 nM), and Y‐27632 (10 µM; Taosu Biotechnology). Culture medium was changed every 2–3 days, and passaging was carried out with TrypLE Express (Gibco). Organoids at passages P1–P4 were used for all experiments.

In the immunofluorescence assay, organoids were first fixed, permeabilised in 0.5% Triton X‐100, and blocked with 5% BSA. They were then incubated with rabbit anti‐KAT2A antibody (1:200, Proteintech), followed by Alexa Fluor 488‐conjugated goat anti‐rabbit IgG (1:500, Thermo Fisher Scientific). DAPI was applied for nuclear visualisation. Images were acquired on a Leica SP8 confocal microscope, and signal quantification was conducted in ImageJ.

### 2.6 Chemosensitivity assay

The chemosensitivity of cells was determined by CCK‐8 assay. Patient‐derived organoids from four independent lines (P1–P4) were dissociated into single cells and seeded at 5 × 10^3^ cells per well in Corning 96‐well ultra‐low attachment plates with 100 µL of Matrigel‐containing culture medium diluted at 1:2. For comparison, PC3 and DU145 cell lines were plated in regular 96‐well plates at 3 × 10^3^ cells per well. After allowing 24 h for recovery, cells were incubated with either cisplatin or docetaxel (MedChemExpress) for 72 h. CCK‐8 reagent (10 µL per well; Dojindo) was then applied, followed by incubation at 37°C for 2 h. Absorbance values at 450 nm were measured on a BioTek Synergy HT microplate reader, and the viability of treated cells was expressed as a percentage of the untreated controls.

### 2.7 Cell culture and transfection

RWPE‐1 human prostate epithelial cells, HEK‐293T cells, and prostate cancer cell lines (PC3, DU145, LNCaP, C4‐2, and 22RV1) were purchased from ATCC. Docetaxel‐resistant PC3 and DU145 cells were obtained commercially (YaJi Biological) and further evaluated in our study by comparison with their parental counterparts under the same drug treatment conditions, confirming their relatively resistant phenotype in the subsequent chemosensitivity assays.

For KAT2A knockout, sgRNAs targeting exons 2–3 were cloned into PX459 vectors and transfected into PC3 and DU145 cells, followed by puromycin selection. For KAT2A overexpression, full‐length coding sequences from PC3/DU145 cDNA were cloned into pCMV‐3×FLAG vectors. Stable cell lines were established by puromycin selection, and expression efficiency was confirmed by Western blotting. Primer and plasmid information is provided in Table S1.

### 2.8 RNA extraction and quantitative real‐time PCR

RNA was prepared from samples using the MolPure TRIeasy Plus Total RNA Kit (YEASEN). Subsequently, 1 µg of total RNA was converted into cDNA with the PrimeScript RT‐PCR Kit (Takara). Quantitative real‐time PCR analysis was conducted with specific primers (Table S2), and transcript abundance was quantified according to the 2^(−ΔΔCt)^ method.

### 2.9 Protein extraction and western blot analysis

Protein extracts were prepared from cells using RIPA lysis buffer (Beyotime) supplemented with protease inhibitors. Protein concentration was determined with the Pierce BCA Protein Assay Kit (Thermo Fisher). Equal amounts of protein were separated by SDS‐PAGE and transferred to PVDF membranes (MilliporeSigma). After blocking, membranes were incubated overnight at 4°C with primary antibodies against KAT2A (1:1000, Proteintech), BBC3 (1:1500, Proteintech) and PIK3R2 (1:2000, Thermo Fisher), followed by 1 h incubation with HRP‐conjugated secondary antibodies. Signals were visualised by enhanced chemiluminescence.

### 2.10 Cell proliferation and apoptosis assays

Colony formation assay: Cells were seeded into six‐well plates at a low density and maintained for 10–14 days to allow colony growth. The resulting colonies were fixed with 4% paraformaldehyde, stained using 0.1% crystal violet, and subsequently quantified with ImageJ software.

EdU incorporation assay: Cell proliferative activity was assessed with the BeyoClick EdU Kit (Beyotime). After incubation with EdU for 2 h, cells were fixed, permeabilised, and subjected to Click chemistry labelling. Hoechst 33342 was used for nuclear staining, and the proportion of EdU‐labelled cells was determined with a Leica SP8 confocal microscope.

TUNEL assay: Apoptotic cells in FFPE xenograft tissue sections were identified using the Beyotime TUNEL Kit. After deparaffinisation and rehydration, the sections underwent antigen retrieval and permeabilisation, followed by incubation with TdT enzyme and labelled dUTP for 1 h at 37°C. Nuclei were counterstained with DAPI, and TUNEL‐positive cells were visualised and quantified on a Leica SP8 confocal microscope.

### 2.11 Xenograft tumour model

Animal studies were performed in accordance with institutional regulations for laboratory animal care. Protocols were approved by the Animal Welfare and Ethics Committee of the Institute of Health and Medicine, Hefei Comprehensive National Science Center (Approval No. IHM‐AP‐2024‐046). Male BALB/c nude mice aged 4–6 weeks were subcutaneously injected with 1 × 10^6^ PC3 cells carrying stable KAT2A knockout (KO) or overexpression (OE) constructs. After tumours became palpable, mice received intraperitoneal docetaxel (10 mg/kg),^25^ cisplatin (5 mg/kg),^26^ or Butyrolactone 3 (MB‐3, 10 mg/kg)^27^ every 3 days for three weeks. Body weight and tumour growth were measured at 3‐day intervals. Tumour volume was determined according to the formula *V* = 0.5 × length × width^2^. Upon completion of the study, mice were euthanised and tumours were excised for downstream analyses.

### 2.12 PIK3R2 protein stability and degradation pathway analysis

PIK3R2 protein stability was evaluated by treating cells with cycloheximide (CHX, 100 µg/mL, Cayman) to block newly synthesised protein production, followed by collection of lysates at the specified time points for Western blotting. To investigate the mechanism of protein turnover, cells were also incubated for 4 h with MG132 (10 µM, Sigma), a proteasome inhibitor, or chloroquine (CQ, 25 µM, MedChemExpress), a lysosomal inhibitor, before analysis.

### 2.13 Immunoprecipitation and post‐translational modification analysis

For immunoprecipitation, cells were lysed on ice with NP‐40 buffer supplemented with protease inhibitors. Protein samples of equal quantity were incubated overnight at 4°C with anti‐PIK3R2 antibody (1:500, Thermo Fisher) and then precipitated using Protein A/G agarose beads. Following extensive washing, immune complexes were released in 1× SDS sample buffer. Post‐translational modifications of PIK3R2, including acetylation, succinylation, and ubiquitination, were examined by immunoblotting with antibodies specific for acetylation (1:1000, Proteintech), succinyl‐lysine (1:1000, Beyotime), and ubiquitin (1:1000, Proteintech). Whole‐cell lysate samples were used as input controls.

### 2.14 Statistical analysis

All in vitro functional experiments were independently repeated at least three times (biological replicates), unless otherwise specified. Patient‐derived organoid experiments were repeated at least twice using histopathologically confirmed PCa specimens. All image‐based quantifications were independently assessed by two blinded reviewers. Data are presented as mean ± SD. Statistical analyses were performed in GraphPad Prism 10.1.2 using unpaired two‐sided Student's *t*‐test or two‐way ANOVA with Tukey's post hoc test, as appropriate. Statistical significance was set at *p* < 0.05.

## 3 RESULTS

### 3.1 Succinylation‐related transcriptomic features and prognosis in prostate cancer

**FIGURE 1:**
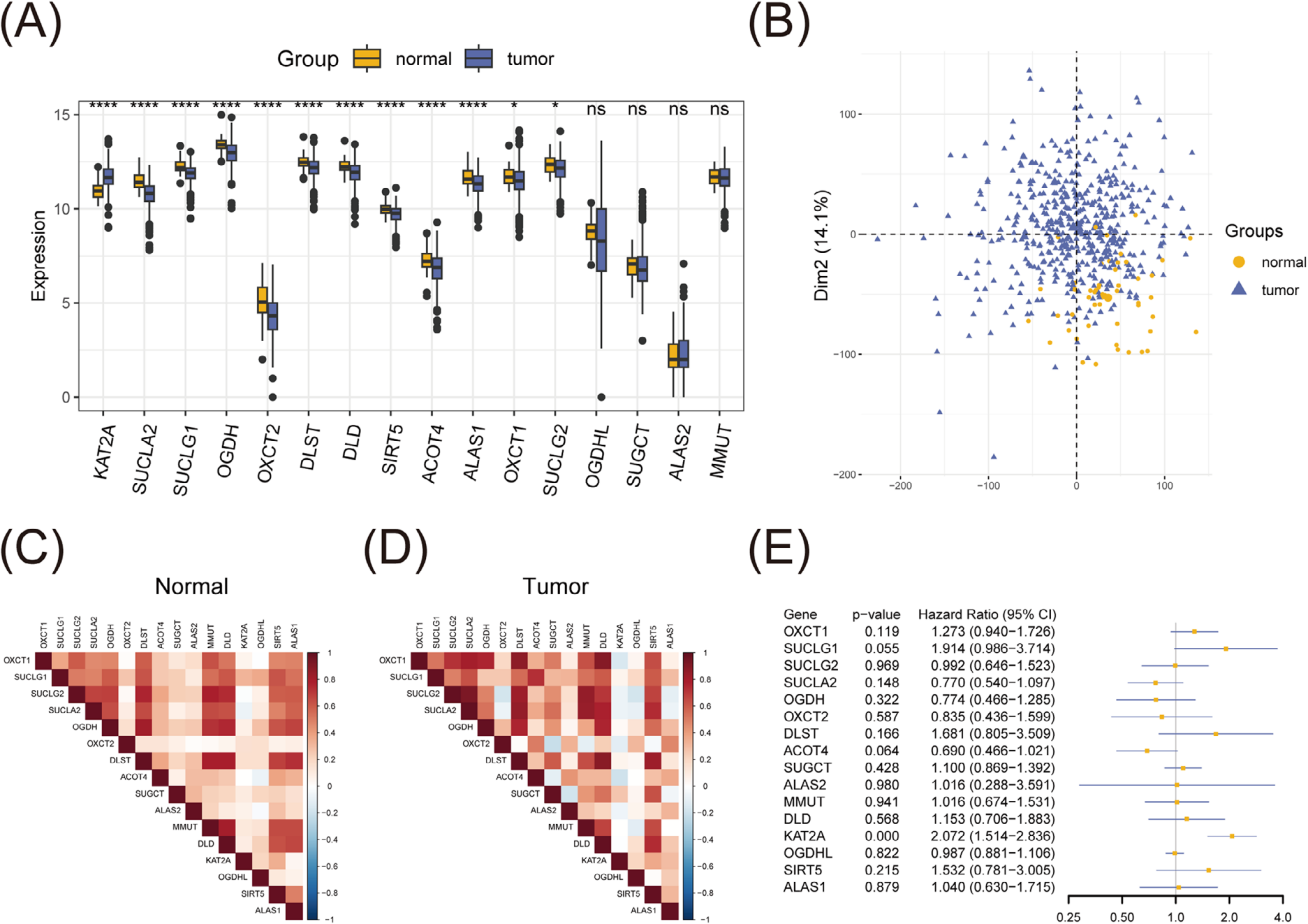
Succinylation‐related transcriptomic features and prognosis in prostate cancer. (A) Boxplots showing the expression levels of succinylation‐related genes in tumour and normal samples from the TCGA‐PRAD cohort. (B) Principal component analysis based on the expression of succinylation‐related genes, comparing tumour and normal tissues. (C, D) Heatmaps of pairwise gene–gene correlations among succinylation‐related genes in normal (C) and tumour (D) samples. (E) Forest plot of univariate Cox regression results for each succinylation‐related gene in relation to biochemical recurrence‐free survival.

Interrogation of TCGA transcriptomic data revealed clear alterations in succinylation‐related gene expression between prostate tumours and adjacent normal tissues. Most enzymes associated with succinylation metabolism,^28^ such as SUCLA2, SUCLG1, OGDH, OXCT2, DLST, DLD, SIRT5, ACOT4, ALAS1, OXCT1, and SUCLG2, were significantly downregulated in tumours, whereas the succinyltransferase KAT2A was markedly upregulated (Figure 1A). This inverse pattern suggests a dysregulated succinylation‐related balance in prostate tumours. Principal component analysis clearly separated tumour from normal samples based on the expression of these genes (Figure 1B), supporting their utility in distinguishing malignant tissues. Co‐expression analysis further demonstrated that the strong positive correlations observed among several succinylation‐related gene pairs in normal tissues were substantially weakened or lost in tumour tissues (Figure 1C and D), indicating a tumour‐specific rewiring of transcriptional networks. Univariate Cox regression analysis identified KAT2A as the only gene significantly associated with poor prognosis (HR > 1, *p* < 0.05), whereas the majority of other succinylation‑related genes did not reach statistical significance in this analysis (Figure 1E). This finding highlights the potential clinical relevance of KAT2A among succinylation regulators in prostate cancer.

To evaluate the generalisability of our findings beyond the TCGA‐PRAD cohort, we performed validation analyses in multiple independent treatment‐naïve prostate cancer datasets. In the GSE21034 and GSE46602 cohorts, the expression patterns of succinylation‐related genes were consistent with those observed in TCGA‐PRAD, with KAT2A significantly upregulated in tumour tissues while most other succinylation‐related enzymes showed downregulation or no significant change (Figure S1A and B). Furthermore, prognostic evaluation in the GSE70769 and GSE116918 cohorts demonstrated that patients with high SS had significantly better biochemical recurrence‐free survival compared to those with low SS (Figure S1C and D). Clinical correlation analyses in these independent cohorts further confirmed that high SS was significantly associated with lower Gleason grade (≤7 vs. ≥8) and earlier tumour stage (T1–T2 vs. T3) (Figure S1E–H), reinforcing the conclusion that high SS identifies less aggressive prostate tumours with favourable prognosis.

### 3.2 Succinylation score stratifies prostate tumours with distinct molecular, clinical, and immune features

**FIGURE 2:**
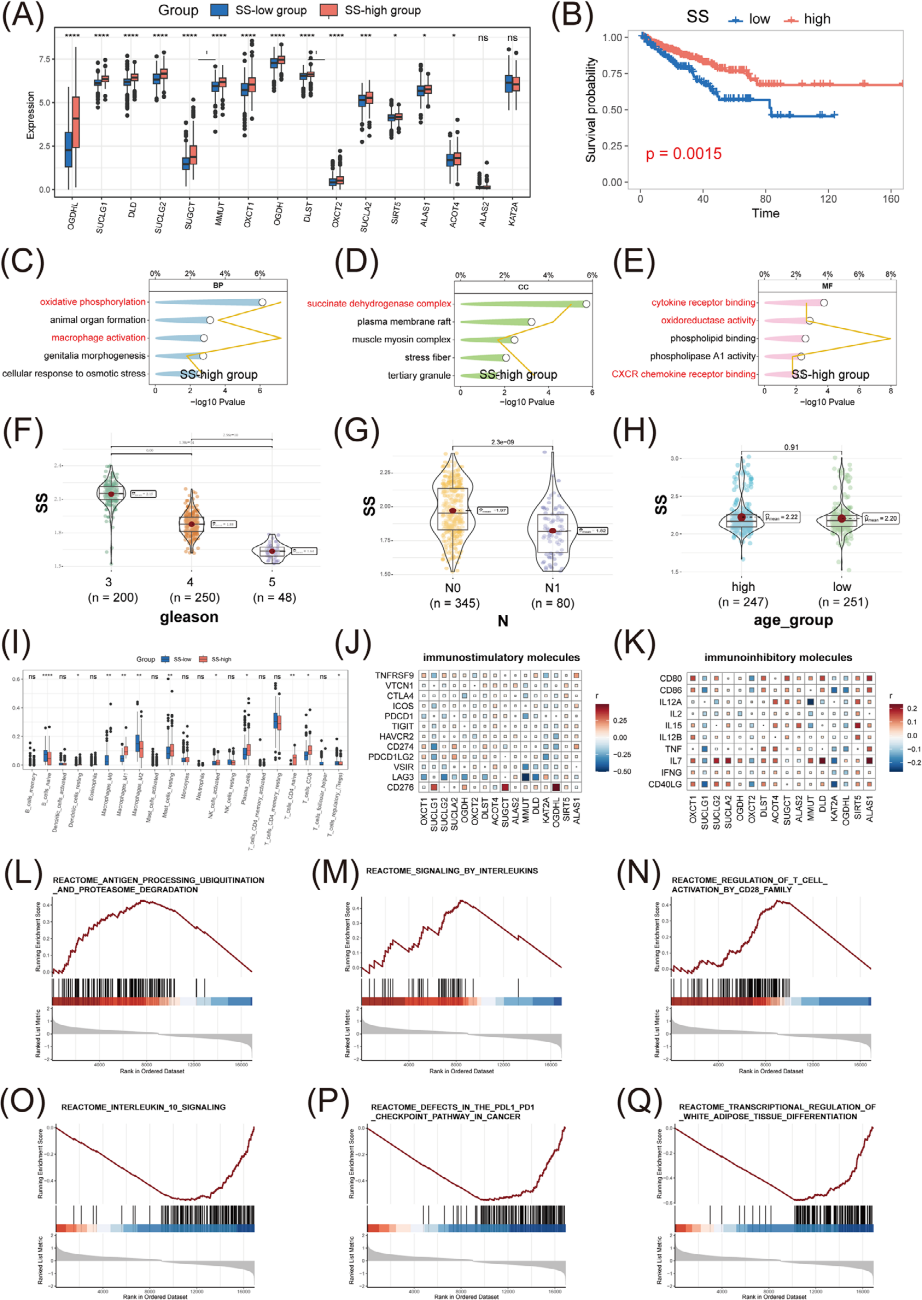
Succinylation score stratifies prostate tumours with distinct molecular, clinical, and immune features. (A) Boxplots comparing expression of succinylation‐related genes between high‐SS and low‐SS groups. (B) Kaplan–Meier curve of biochemical recurrence‐free survival stratified by high‐SS and low‐SS groups. (C–E) GO enrichment results for biological process (C), cellular component (D), and molecular function (E) categories in the high‐SS group. (F–H) Distribution of succinylation scores across Gleason scores (F), lymph node status (G), and age groups (H). (I) Boxplots showing immune cell infiltration levels in high‐SS and low‐SS groups. (J) Correlation heatmap between succinylation‐related genes and immunostimulatory molecules. (K) Correlation heatmap between succinylation‐related genes and immunoinhibitory molecules. (L–Q) GSEA enrichment plots for selected immune‐related pathways between high‐SS and low‐SS groups.

A succinylation score was established by ssGSEA to reflect the overall activity of the succinylation pathway. Based on the median value, patients were divided into high‐SS and low‐SS subgroups. The majority of succinylation‐related genes were more highly expressed in the high‐SS subgroup, while KAT2A expression remained largely unchanged between the two groups (Figure 2A), which supports the robustness of the scoring approach. Survival analysis showed that the high‐SS subgroup had significantly better biochemical recurrence‐free survival (BCRFS; Figure 2B). BCRFS was used as the primary endpoint due to the limited number of overall survival events in this cohort. Functional enrichment analysis showed that the high‐SS group was enriched in processes including oxidative phosphorylation, macrophage activation, and response to osmotic stress (Figure 2C); cellular components such as the succinate dehydrogenase complex and plasma membrane raft (Figure 2D); and molecular functions including cytokine receptor binding and oxidoreductase activity (Figure 2E), indicating broad involvement in metabolism and immune regulation. Clinically, a high‐SS correlated with lower Gleason scores (Figure 2F) and the absence of lymph node metastasis (Figure 2G), but not with patient age (Figure 2H), supporting its role as a marker of less aggressive disease.

High‐SS tumours showed increased infiltration of M1 macrophages, activated NK cells and CD8+ T cells, along with reduced M2 macrophages and Tregs (Figure 2I), consistent with an immunologically activated microenvironment. Succinylation‐related gene expression also positively correlated with multiple co‐stimulatory immune markers (CD274/PD‐L1, ICOS, CD276; Figure 2J), whereas correlations with inhibitory checkpoint molecules were weaker (Figure 2K). GSEA further showed enrichment of antigen‐presentation, interleukin‐signalling, and T‐cell‐activation pathways in high‐SS tumours (Figure 2L–N). By contrast, low‐SS tumours were enriched for pathways associated with immune dysfunction, including IL‐10 signalling and PD‐1 checkpoint defects (Figure 2O–Q). Together, these results connect higher succinylation activity with stronger immune activation and improved antigen‐presentation capacity.

Differential expression analysis between tumour and normal tissues identified several dysregulated genes (e.g., upregulation of LMNTD2‐AS1, PDIA2, WDR92, downregulation of SLC5A8, RPE65, ORM1; Figure S2A). Comparison of high‐SS vs. low‐SS groups revealed a more extensive set of differentially expressed genes (DEGs), including upregulation of immune/metabolic genes (CXCL10, IRF7, SDHB, IDH3A) and downregulation of metabolic suppressors (SCD, PAD1, PPARGC1A) in the high‐SS group (Figure S2B). Unsupervised clustering showed clear separation between tumour/normal and high‐SS/low‐SS groups, with the latter exhibiting more distinct transcriptomic divergence (Figure S2C and D), suggesting that succinylation score is a robust classifier of tumour heterogeneity.

### 3.3 Single‐cell analysis reveals cellular basis of succinylation activity and identifies associations with therapy response

Having established that high‐SS tumours display a more immunostimulatory microenvironment and greater predicted sensitivity to chemotherapy, we next asked whether these transcriptomic differences could be resolved at single‐cell resolution and whether low‐SS tumours harbour specific malignant subpopulations enriched for resistance‐associated drivers.

To assess how succinylation activity shapes the TME at single‐cell resolution, we analysed scRNA‐seq data from the GSE137829 dataset. For each patient, gene expression profiles were aggregated by pseudobulk averaging, and a succinylation score was calculated using ssGSEA based on the predefined succinylation‐related gene set. Patients were then divided into SS‐high and SS‐low groups according to the median score, and all single cells were annotated accordingly. A UMAP embedding of all cells revealed the composition of the TME, including malignant epithelial cells, fibroblasts, endothelial cells, myeloid cells, T and NK cells, and B cells (Figure S3A). When stratified by SS score, the SS‐low group showed a clear dominance of malignant cells, whereas the SS‐high group exhibited greater proportions of immune (T/NK, myeloid) cell populations (Figure S3B and C).

**FIGURE 3:**
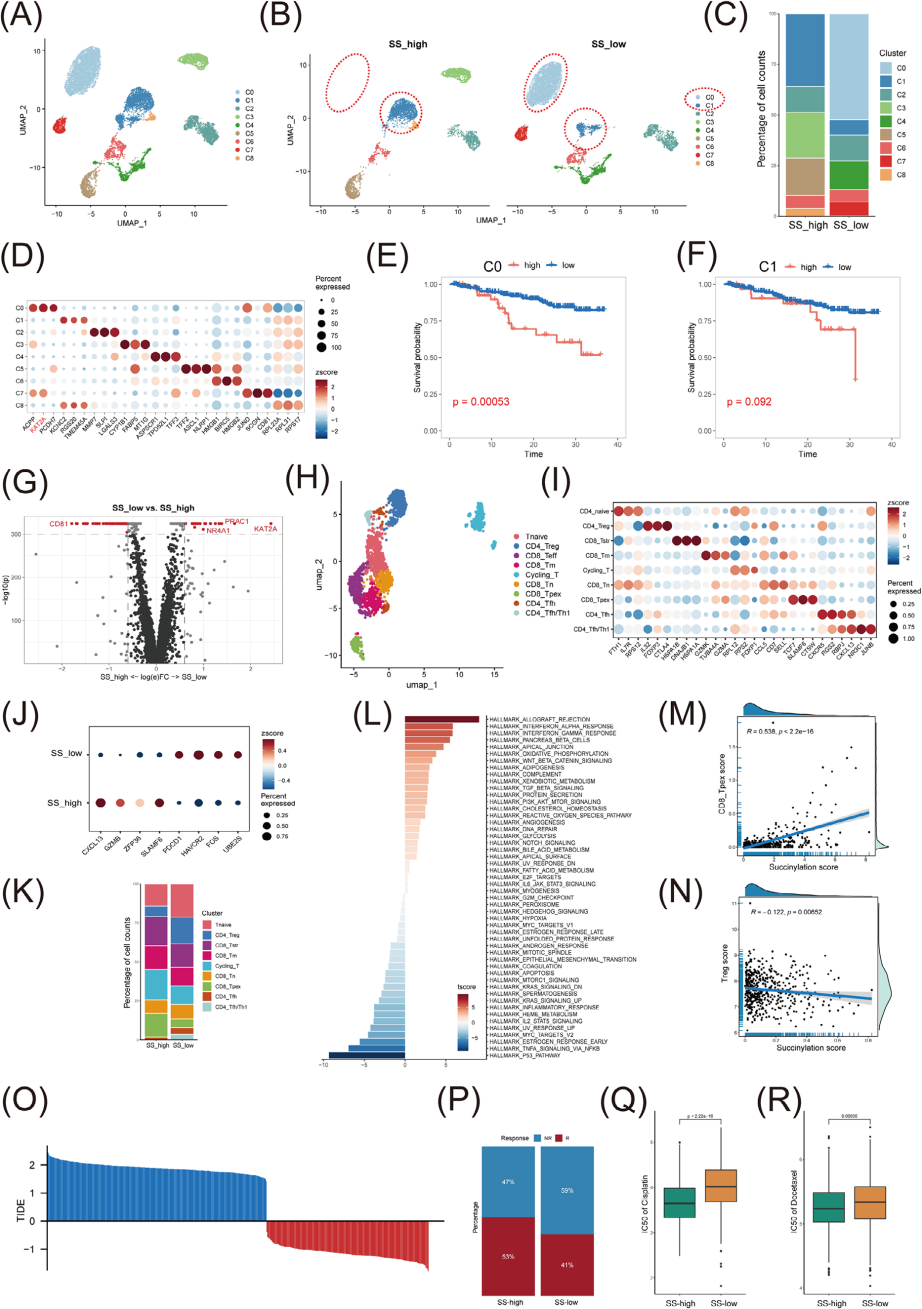
Single‐cell analysis reveals cellular basis of succinylation activity and predicts therapy response. (A) UMAP plot of malignant epithelial cells clustered into nine subgroups (C0–C8). (B) UMAP plots of malignant cells stratified by high‐SS and low‐SS groups. (C) Barplot showing cluster distribution across high‐SS and low‐SS groups. (D) Dotplot showing representative marker genes for each malignant cluster. Dot size indicates the percentage of cells expressing the gene (per cent expressed, 0–100%); color intensity indicates the average normalised expression level. (E, F) Kaplan–Meier curves comparing biochemical recurrence‑free survival between patients with high vs. low enrichment of C0 (E) and C1 (F) clusters, stratified by the median proportion of each cluster among total malignant cells. (G) Volcano plot of DEGs between malignant cells from high‐SS and low‐SS groups. (H) UMAP plot of T cells clustered into distinct subsets. (I) Dotplot showing expression of key marker genes across T cell subsets. (J) Dotplot comparing Tpex marker expression between high‐SS and low‐SS groups. (K) Barplot of T cell subset composition in high‐SS and low‐SS groups. (L) Pathway enrichment analysis based on CD8^+^ Tpex expression profiles. (M, N) Correlation between succinylation score and Tpex (M) or Treg (N) signature scores in TCGA‐PRAD samples. (O) Distribution of TIDE scores for patients in high‐SS and low‐SS groups. (P) Proportion of predicted ICB responders (R) and non‐responders (NR) in each group. (Q, R) Predicted IC_50_ values for cisplatin (Q) and docetaxel (R) in high‐SS and low‐SS tumours based on oncoPredict.

To further dissect tumour‐intrinsic heterogeneity, malignant epithelial cells were re‐clustered into nine transcriptionally distinct subpopulations (C0‐C8), each defined by characteristic marker gene signatures (Figure 3A and D). Among them, C0 was the most abundant cluster in the low‐SS group, whereas C1 predominated in the high‐SS group (Figure 3B and C). Importantly, further examination of patient‐level contributions revealed that C0 cells were enriched across the majority of SS_low patients rather than being driven by one or a few individual samples, supporting the robustness of this observation. Notably, the SS‐low‐enriched cluster C0 exhibited gene expression patterns associated with stress response, metabolism, and immune evasion, suggesting a more aggressive transcriptional state. Specifically, C0 cells showed significant upregulation of stress‐responsive genes (e.g., *JUN*, *FOS*), metabolic regulators (e.g., *ENO1*, *LDHA*), and immune‐modulatory factors (e.g., *TGFB1*, *IL6*) compared to other malignant clusters, supporting these functional interpretations. Kaplan–Meier survival analysis showed that patients enriched in C0 had significantly poorer BCRFS, while C1 dominance showed a relatively favourable trend (Figure 3E and F). Given that the above scRNA‐seq analysis was performed on CRPC samples, we sought to validate these findings in a treatment‐naïve primary prostate cancer scRNA‐seq dataset (GSE141445) to ensure biological consistency across disease stages. Analysis of cell‐type composition in GSE141445 revealed that the SS‐low group was predominantly composed of malignant cells, whereas the SS‐high group exhibited a higher proportion of immune cell populations, including T/NK and myeloid cells (Figure S3G–I). Re‐clustering of malignant epithelial cells identified distinct subpopulations, with the SS‐low group enriched in a specific malignant cluster (C0) and the SS‐high group enriched in another cluster (C3) (Figure S3D–F). Notably, *KAT2A* expression was significantly elevated in malignant cells from the SS‐low group compared to those from the SS‐high group (Figure S3J), consistent with our observations in the CRPC dataset. These findings confirm that *KAT2A* upregulation in low‐SS malignant cells is a conserved feature across both treatment‐naïve and castration‐resistant prostate cancer, supporting the robustness of our single‐cell‐based conclusions. DEG analysis identified upregulation of KAT2A, PRAC1 and NR4A1 in low‐SS malignant cells (Figure 3G), further linking low‐SS to aggressive tumour phenotypes.

T cell clustering identified multiple subsets, including CD8^+^ Tpex cells, which were markedly enriched in high‐SS tumours (Figure 3H–K). Pathway analysis showed that Tpex cells were associated with interferon signalling, allograft rejection, and IL‐2/STAT5 activation (Figure 3L). In the TCGA cohort, SS positively correlated with CD8^+^ Tpex signature scores and showed a modest inverse correlation with Treg signatures (Figure 3M and N), reinforcing the link between high succinylation and an immunostimulatory TME.

TIDE analysis indicated a higher proportion of predicted ICB responders in the high‐SS group (Figure 3O and P). Using oncoPredict, we found that high‐SS tumours had lower predicted IC_50_ values for cisplatin and docetaxel (Figure 3Q and R), suggesting a potential association with enhanced chemosensitivity. It should be noted that these in silico predictions are exploratory and hypothesis‐generating; they do not substitute for direct clinical validation.

### 3.4 KAT2A expression is elevated in tumours and correlates with chemoresistance in prostate cancer

Given the upregulation of KAT2A in low‐SS prostate tumours (Figure 3G) and its high expression within the low‐SS enriched malignant subcluster C0 (Figure 3B and D), we hypothesised that KAT2A may act as a potential oncogenic driver in the context of reduced succinylation activity. To validate this hypothesis, we evaluated KAT2A expression and chemoresistance phenotypes using patient‐derived clinical samples and organoid models.

**FIGURE 4:**
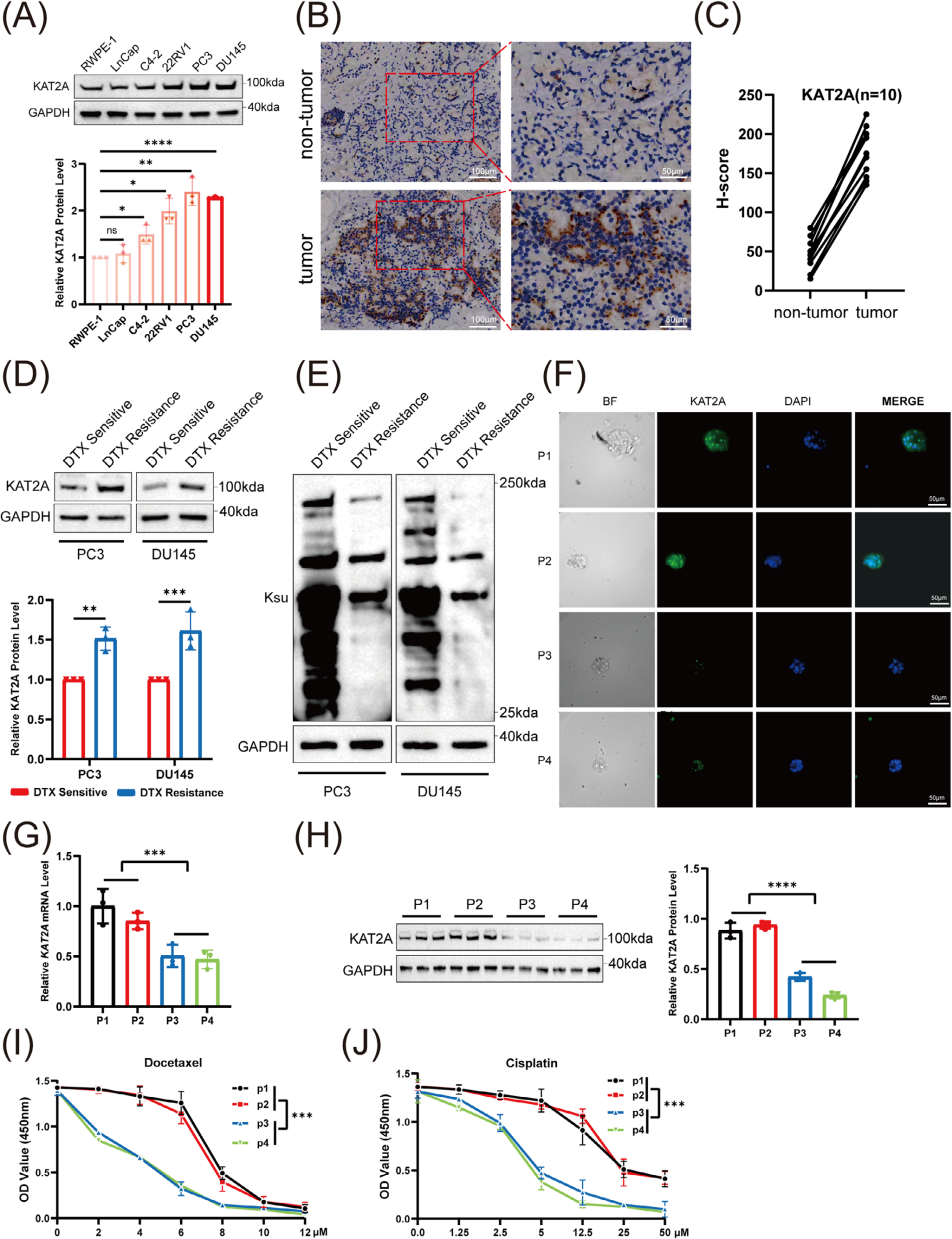
Validation of differential KAT2A expression and chemotherapy response in vitro and in vivo. (A) Western blot analysis of KAT2A protein levels in a panel of prostate cell lines, including the normal epithelial line RWPE‐1 and prostate cancer cell lines LNCaP, C4‐2, 22RV1, PC3, and DU145. Lower panel shows densitometric quantification of protein bands. (B) Representative IHC staining of KAT2A in tumour tissues and matched adjacent non‐tumour prostate tissues. (C) H‐score quantification of KAT2A expression in paired tumour and non‐tumour samples (*n* = 10). (D) Western blot comparison of KAT2A protein levels between parental and docetaxel‐resistant PC3 and DU145 cell lines. Lower panel shows densitometric quantification. (E) Western blot comparison of succinylation level between parental and docetaxel‐resistant PC3 and DU145 cell lines. (F) Representative immunofluorescence staining for KAT2A in patient‐derived organoids from four individuals (P1–P4), showing bright‐field (BF), KAT2A signal, DAPI nuclear counterstain, and merged images. (G) Quantitative real‐time PCR analysis of *KAT2A* mRNA levels in patient‐derived organoids (P1–P4). (H) Western blot analysis of KAT2A protein levels in patient‐derived organoids (P1–P4). Right panel shows densitometric quantification. (I) Dose–response curves for patient‐derived organoids (P1–P4) treated with docetaxel. (J) Dose–response curves for patient‐derived organoids (P1–P4) treated with cisplatin. Data are presented as mean ± SD. Statistical significance was defined as follows: *p < 0.05, **p < 0.01, ***p < 0.001, ****p < 0.0001; ns indicates not significant.

We first compared KAT2A expression between prostate epithelial cells and prostate cancer cells, and found that KAT2A was markedly upregulated in tumour cells. Moreover, its expression increased with tumour malignancy grade (Figure 4A). Immunohistochemical staining revealed stronger KAT2A signals in tumour tissues compared with matched adjacent non‐tumour regions, with predominant nuclear localisation (Figure 4B). H‐score quantification confirmed significantly elevated KAT2A expression in tumour areas across ten patient samples (Figure 4C). To further explore KAT2A expression in chemoresistant prostate cancer cells, we used commercially obtained docetaxel‐resistant PC3 and DU145 cell lines, which exhibited increased KAT2A expression relative to their parental counterparts (Figure 4D). Notably, the global protein succinylation level was reduced in the docetaxel‐resistant cell lines (Figure 4E). We next investigated potential functional variation in prostate cancer organoids derived from four patients. Immunofluorescence staining showed that organoids from patients P1 and P2 (KAT2A‐high) displayed strong nuclear KAT2A signals, whereas organoids from patients P3 and P4 (KAT2A‐low) exhibited weak or undetectable signals (Figure 4F). PCR and Western blot analyses further validated the differential KAT2A mRNA and protein expression between these patient‐derived organoids (Figure 4G and H). Finally, chemotherapeutic response was assessed in these organoids. Cell viability assays following cisplatin or docetaxel exposure demonstrated that KAT2A‐high organoids maintained significantly higher viability compared with KAT2A‐low organoids at increasing drug concentrations (Figure 4I and J). Collectively, these findings indicate that KAT2A acts as an oncogenic effector driving chemoresistance in succinylation‐deficient prostate tumours.

### 3.5 KAT2A regulates chemosensitivity under drug stress

**FIGURE 5:**
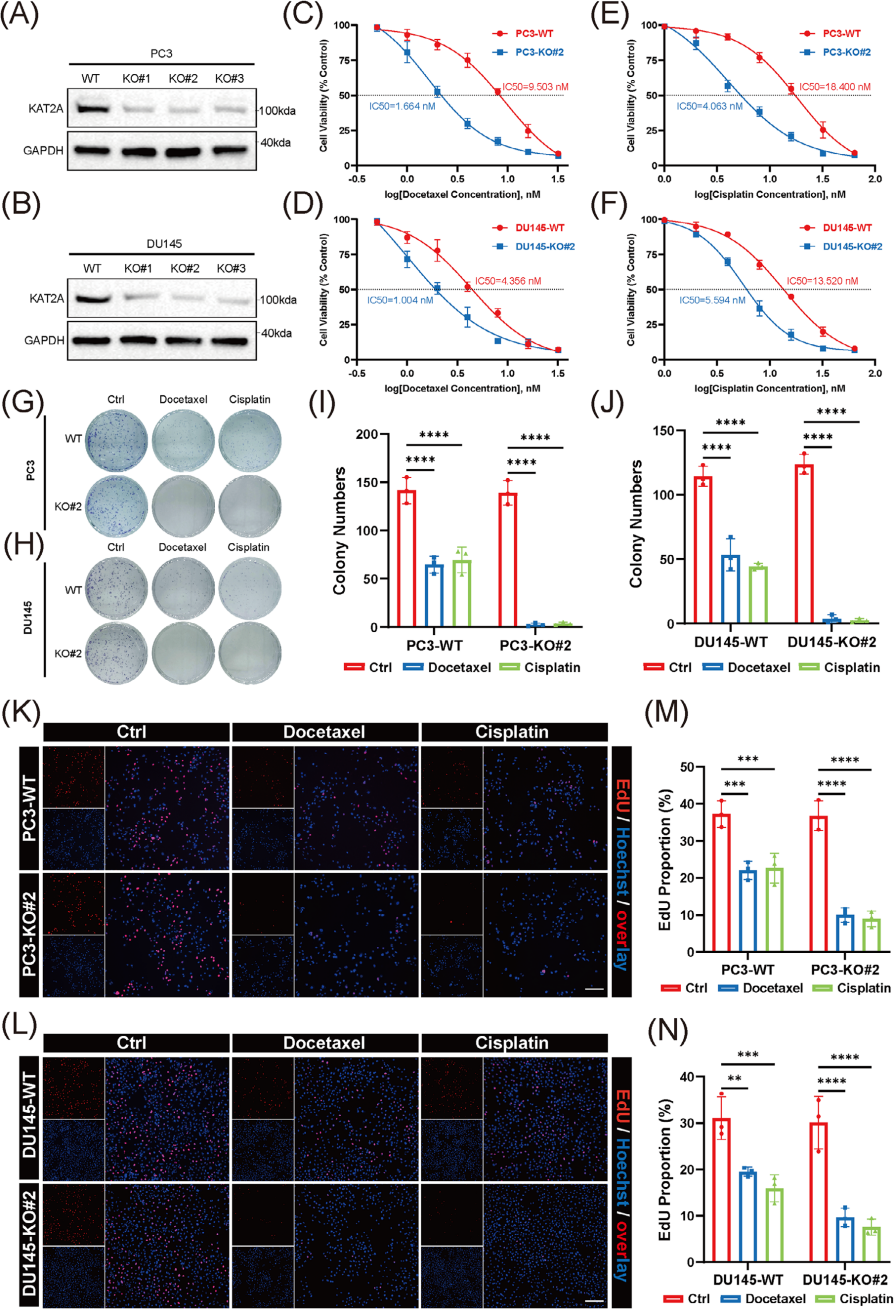
Knockout of KAT2A sensitises prostate cancer cells to docetaxel and cisplatin treatment in vitro. (A, B) Western blot validation of KAT2A knockout efficiency in PC3 (A) and DU145 (B) cells. (C, D) Determination of the IC_50_ for docetaxel in PC3 (C) and DU145 (D) cells following KAT2A knockout, measured using the CCK‐8 assay. (E, F) Determination of IC_50_ values for cisplatin in PC3 (E) and DU145 (F) cells following KAT2A knockout, measured using the CCK‐8 assay. (G, H) Representative images of colony formation assays for PC3 (G) and DU145 (H) cells after KAT2A knockout. (I, J) Quantification of colonies formed by PC3 (I) and DU145 (J) cells. (K, L) Representative EdU incorporation assay images for PC3 (K) and DU145 (L) cells after KAT2A knockout. Scale bar: 100 µm. (M, N) Quantification of EdU‐positive cells in PC3 (M) and DU145 (N) populations. Data are presented as mean  ±  SD. Statistical significance: *p  <  0.05; **p  <  0.01; ***p  <  0.001; ****p  <  0.0001.

To investigate the role of KAT2A in regulating chemosensitivity of prostate cancer cells, we first knocked out KAT2A in PC3 and DU145 cell lines and verified the knockout efficiency via Western blot analysis (Figure 5A and B). Results showed that KO#2 sample exhibited optimal knockout efficiency and was therefore selected for subsequent functional experiments. Drug sensitivity assays demonstrated that KAT2A deletion significantly reduced the IC_50_ values for docetaxel in both cell lines (Figure 5C and D), indicating enhanced cellular sensitivity to this drug. Cisplatin treatment also revealed significantly decreased IC_50_ values following KAT2A knockout in both cell lines (Figure 5E and F), further supporting that KAT2A deficiency enhances chemotherapeutic drug efficacy.

In functional assays, colony formation experiments showed that under chemotherapeutic drug treatment, the KAT2A‐deficient group exhibited significantly reduced colony numbers (Figure 5G–J), indicating impaired survival and proliferative capacity under drug stress. EdU incorporation assays further supported this finding: following chemotherapeutic drug treatment, the proportion of proliferating cells in the KAT2A‐deficient group was significantly lower than in the control group (Figure 5K–N). These results collectively suggest that KAT2A deletion reduces tumour cell survival and proliferative capacity under drug pressure, thereby enhancing chemosensitivity. Notably, combined analysis of colony formation and EdU results revealed that under drug‐free conditions, KAT2A deletion had limited effects on basal proliferation and colony formation capacity, indicating that KAT2A primarily regulates the chemotherapy response rather than basal cell growth.

Conversely, KAT2A overexpression experiments in PC3 and DU145 cells were similarly validated by Western blot (Figure S4A and B). In contrast to knockout results, KAT2A overexpression significantly increased IC_50_ values for both docetaxel (Figure S4C and D) and cisplatin (Figure S4E and F), suggesting reduced chemosensitivity. Further colony formation assays demonstrated that even under chemotherapeutic drug treatment, KAT2A‐overexpressing cells could form more colonies (Figure S4F–J). EdU probe incorporation assays similarly showed that in the presence of drugs, the KAT2A‐overexpressing group exhibited higher proliferation rates than the control group (Figure S4K–N). These results indicate that KAT2A overexpression maintains or even enhances prostate cancer cell growth and survival under drug stress, manifesting as significant chemoresistance.

### 3.6 KAT2A promotes chemoresistance in prostate cancer by regulating PIK3R2 succinylation

Given the strong association between KAT2A and chemoresistance phenotypes, we next investigated the molecular mechanism by which KAT2A promotes drug resistance, with particular focus on post‐translational regulation of downstream effector proteins.

**FIGURE 6:**
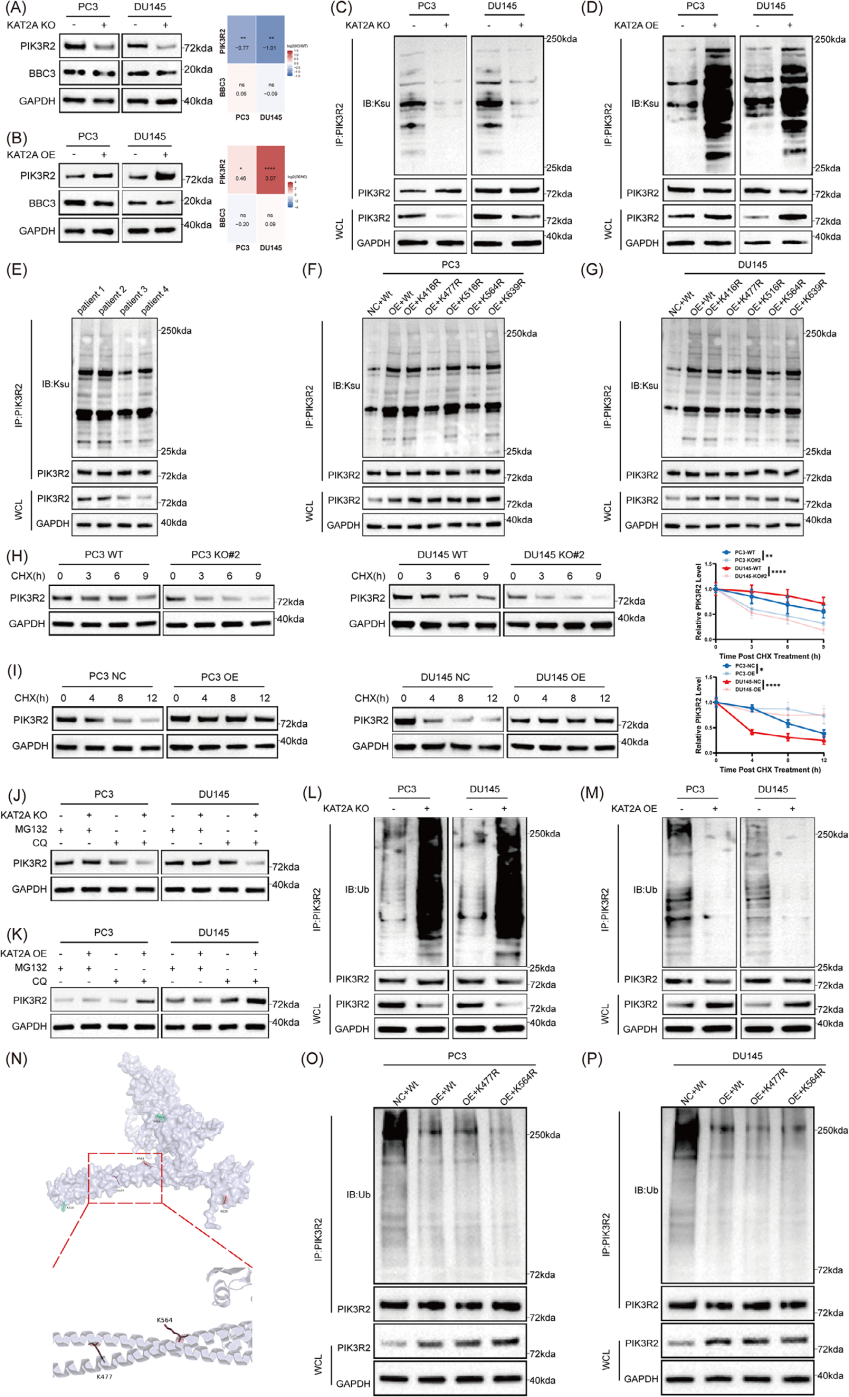
KAT2A promotes chemoresistance in prostate cancer by regulating PIK3R2 succinylation. (A, B) Western blot analysis of PIK3R2 and BBC3 protein levels following KAT2A knockout (A) or overexpression (B). Right panels show densitometric quantification of protein bands. (C) Detection of PIK3R2 succinylation after KAT2A knockout by immunoprecipitation using an anti‐PIK3R2 antibody, followed by immunoblotting with an anti‐succinyl‐lysine antibody. (D) Detection of PIK3R2 succinylation after KAT2A overexpression using the same immunoprecipitation and immunoblot workflow. (E) PIK3R2 succinylation levels in human prostate cancer organoids. (F, G) Site‐directed mutagenesis of candidate PIK3R2 succinylation residues (K‐to‐R mutants) and analysis of PIK3R2 succinylation in PC3 (F) and DU145 (G) cells. (H) Cycloheximide (CHX) chase assay of PIK3R2 protein stability after KAT2A knockout. Cells were treated with CHX for 0, 3, 6, and 9 h, and PIK3R2 protein levels were analysed by Western blot. Right panel shows densitometric quantification. (I) CHX chase assay of PIK3R2 protein stability after KAT2A overexpression. Cells were treated with CHX for 0, 4, 8, and 12 h; PIK3R2 protein levels were determined by Western blot. Right panel shows densitometric quantification. (J, K) Western blot analysis of PIK3R2 protein levels following KAT2A knockout (J) or overexpression (K), in the presence or absence of the proteasome inhibitor MG‐132 or lysosomal inhibitor chloroquine (CQ). (L) Detection of PIK3R2 ubiquitination after KAT2A knockout by immunoprecipitation with an anti‐PIK3R2 antibody and immunoblotting using an anti‐ubiquitin antibody. (M) Detection of PIK3R2 ubiquitination after KAT2A overexpression by the same immunoprecipitation and immunoblotting procedures. (N) Structural visualisation of the PIK3R2 three‐dimensional model and the indicated succinylation sites. (O, P) Site‐directed mutagenesis of candidate ubiquitination residues (K‐R) and analysis of PIK3R2 ubiquitination in PC3 (O) and DU145 (P) cells. Data are presented as mean ± SD. Statistical significance was defined as follows: *p < 0.05, **p < 0.01, ***p < 0.001, ****p < 0.0001; ns indicates not significant.

We first screened chemoresistance‐related pathways using the MSigDB resource. Pathway enrichment showed that DNA damage response pathways were most strongly enriched in the SS‐low group (Figure S5A). The top 30 genes from this pathway are displayed in Figure S5B, and correlation analysis of the top 10 genes showed that KAT2A expression was positively associated with the resistance‐related genes BBC3 and PIK3R2 (Figure S5C). To further explore KAT2A's regulatory mechanism on downstream target genes, we first examined its effects on transcriptional levels. Real‐time quantitative PCR analysis indicated that KAT2A manipulation did not significantly alter *PIK3R2* and *BBC3* mRNA levels. Under both KAT2A knockout (Figure S5D) and overexpression (Figure S5E) conditions, *PIK3R2* and *BBC3* transcriptional levels remained largely stable, clearly demonstrating that KAT2A regulates PIK3R2 at the post‐transcriptional level rather than through gene expression regulation. However, at the protein level, we observed distinctly different phenomena. Western blot analysis revealed that KAT2A significantly regulates PIK3R2 protein abundance. KAT2A knockout (Figure 6A) led to a dramatic decrease in PIK3R2 protein levels, while KAT2A overexpression (Figure 6B) significantly enhanced PIK3R2 protein expression. Notably, BBC3 protein levels did not exhibit corresponding changes, indicating that KAT2A's regulation of PIK3R2 is specific rather than broadly affecting all related proteins.

Given that KAT2A altered PIK3R2 protein abundance without affecting its mRNA expression, we hypothesised that this regulation may involve post‐translational modifications (PTMs). Because KAT2A is a well‐characterised acetyltransferase, we first tested whether it regulates PIK3R2 via acetylation. DEEPla prediction identified three potential acetylation sites in PIK3R2 (K376, K529, and K584). However, co‐immunoprecipitation assays showed that neither KAT2A knockout nor overexpression significantly changed the acetylation level of PIK3R2 (Figure S5F and G). Consistently, in human organoid models, no correlation was observed between KAT2A expression and PIK3R2 acetylation (Figure S5H), suggesting that acetylation is unlikely to account for KAT2A‐dependent regulation of PIK3R2.

Because KAT2A also possesses succinyltransferase activity, we next investigated whether it regulates PIK3R2 through succinylation. Co‐immunoprecipitation revealed that KAT2A depletion significantly reduced PIK3R2 succinylation (Figure 6C), whereas KAT2A overexpression markedly enhanced this modification (Figure 6D), supporting a specific role for KAT2A as a succinyltransferase for PIK3R2. In human organoids, PIK3R2 succinylation was increased in organoids with high KAT2A expression and decreased in those with low KAT2A expression (Figure 6E).

Because post‐translational modifications often affect protein stability. To determine whether succinylation affects PIK3R2 stability, we performed cycloheximide (CHX) chase assays. KAT2A loss significantly accelerated PIK3R2 degradation (Figure 6H), whereas KAT2A overexpression prolonged its half‐life (Figure 6I). These data indicate that KAT2A‐mediated succinylation is critical for maintaining PIK3R2 protein stability.

To identify the KAT2A‐regulated succinylation sites on PIK3R2, we used GPSuc to predict potential succinylation residues and obtained five candidates: K416, K477, K516, K564, and K639. These sites did not overlap with the predicted acetylation residues, implying that the acetylation and succinylation functions of KAT2A may operate independently. We then generated lysine‐to‐arginine (K→R) mutants to mimic desuccinylation and evaluated their effects. Site‐directed mutagenesis showed that K416R, K516R, and K639R had minimal impact on PIK3R2 succinylation, whereas K477R and K564R markedly reduced succinylation levels. Thus, K477 and K564 are likely the principal residues mediating KAT2A‐dependent PIK3R2 succinylation.

Because succinylation and ubiquitination can compete for modification on lysine residues,^29^ we next examined how KAT2A affects PIK3R2 degradation pathways. Cells were exposed to either the proteasome inhibitor MG132 or the lysosome inhibitor chloroquine (CQ),^30^ we found that changes in PIK3R2 protein levels following KAT2A knockout or overexpression were primarily reversed by MG132 (Figure 6J and K), with limited effects from CQ treatment, indicating that the proteasome pathway is the primary mechanism for KAT2A‐regulated PIK3R2 degradation.

To directly assess the competition between succinylation and ubiquitination, we examined PIK3R2 ubiquitination. Co‐immunoprecipitation showed that KAT2A depletion significantly increased PIK3R2 ubiquitination (Figure 6L), whereas KAT2A overexpression markedly suppressed ubiquitination (Figure 6M). We further investigated whether this competition occurs at K477 and K564. Structural analysis revealed that both residues are exposed on the protein surface, providing spatial accessibility for ubiquitination (Figure 6N). Moreover, K477R and K564R mutations significantly reduced PIK3R2 ubiquitination (Figure 6O and P). These results indicate that K477 and K564 serve as shared target residues for both succinylation and ubiquitination; substitution of lysine with arginine abolishes both modifications, functionally supporting a competitive relationship at these sites.

Collectively, our findings support a model in which KAT2A‐mediated succinylation of PIK3R2 at K477 and K564 functionally antagonises its ubiquitination, thereby reducing proteasome‐dependent degradation and stabilising PIK3R2.

### 3.7 KAT2A promotes in vivo resistance to docetaxel and cisplatin in prostate cancer

**FIGURE 7:**
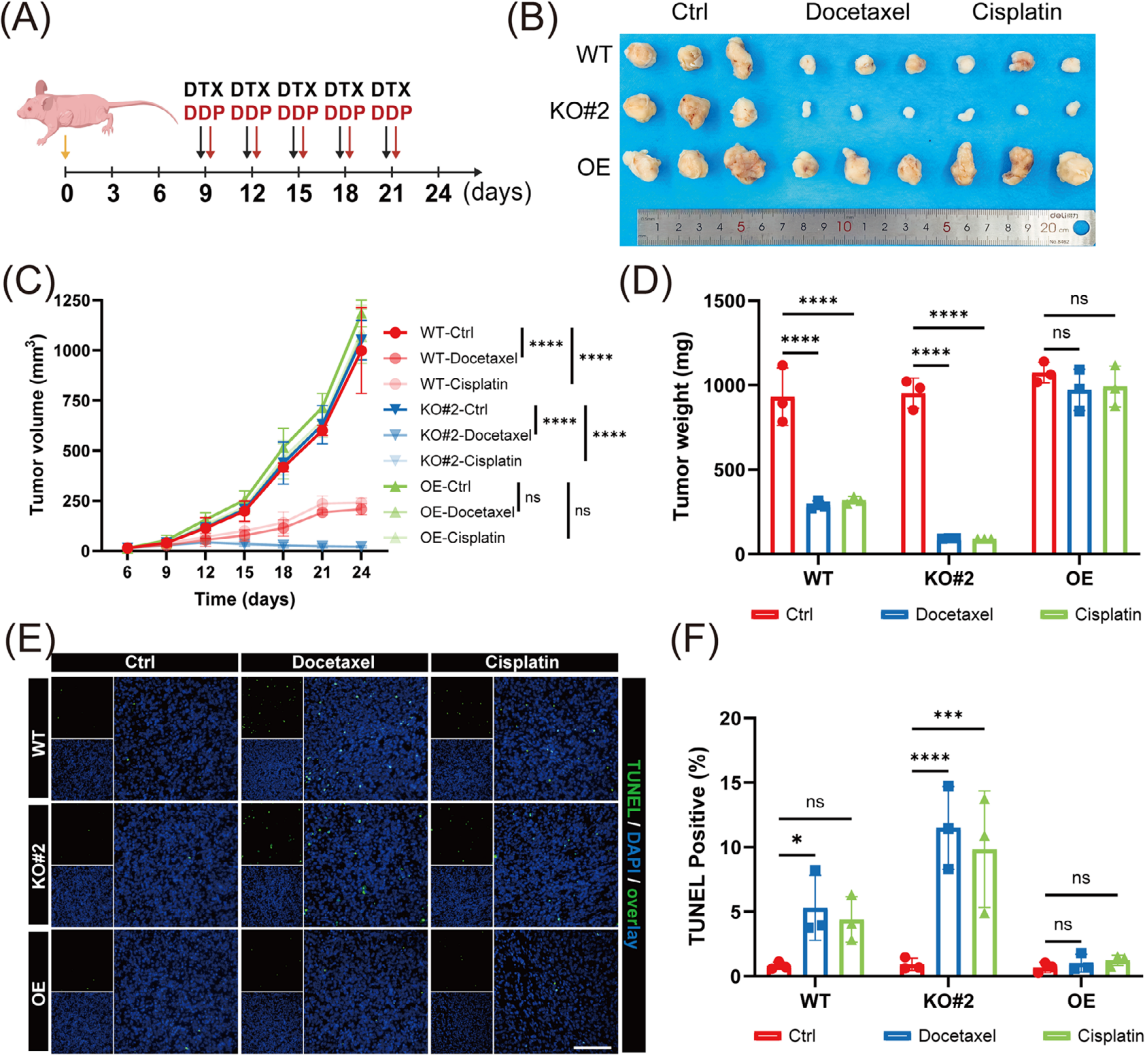
KAT2A promotes docetaxel and cisplatin resistance in prostate cancer in vivo. (A) Schematic diagram illustrating the experimental design for subcutaneous tumour formation in BALB/c nude mice. (B) Representative images of subcutaneous tumours from the control, KAT2A knockout, and KAT2A overexpression groups following treatment with docetaxel and cisplatin. (C) Tumour volume was measured every 3 days, and the corresponding growth curves are presented. (D) Tumour weight upon excision at the endpoint. (E) Representative images of TUNEL staining demonstrating apoptosis in tumour sections after drug treatment. Scale bar: 200 µm. (F) Quantification of TUNEL‐positive cells. Data are presented as mean ± SD. Statistical significance was defined as follows: *p < 0.05, **p < 0.01, ***p < 0.001, ****p < 0.0001; ns indicates not significant.

To determine whether the in vitro findings extend to animals, we generated subcutaneous xenograft models in BALB/c nude mice using PC3 cells with stable KAT2A knockout or overexpression. Once tumours became palpable, mice were treated with docetaxel (10 mg/kg) or cisplatin (5 mg/kg) (Figure 7A). In vivo results demonstrated that KAT2A expression levels significantly determined tumour responsiveness to these two chemotherapeutic drugs. Representative tumour appearances (Figure 7B) and tumour growth curves (Figure 7C) both indicated that under identical chemotherapy conditions, KAT2A knockout group tumours exhibited significantly inhibited growth with much smaller volumes than controls, while KAT2A overexpression group tumours showed significantly increased volumes, displaying stronger resistance phenotypes. Tumour weight measurements at experimental endpoints were consistent with volume changes (Figure 7D), further supporting these conclusions. To investigate the cytological mechanisms, we performed TUNEL staining on tumour tissues to assess apoptosis. Results showed significantly increased TUNEL‐positive cells in KAT2A knockout group tumours, while KAT2A overexpression group exhibited significantly reduced apoptotic cells (Figure 7E), with quantitative analysis supporting this trend (Figure 7F). These data indicate that KAT2A may promote tumour cell tolerance to docetaxel and cisplatin in vivo by inhibiting chemotherapy‐induced apoptosis; conversely, KAT2A loss enhances chemotherapy‐associated cell death, thereby suppressing tumour growth.

### 3.8 KAT2A inhibitor MB‐3 enhances docetaxel and cisplatin efficacy in vitro and in vivo

**FIGURE 8:**
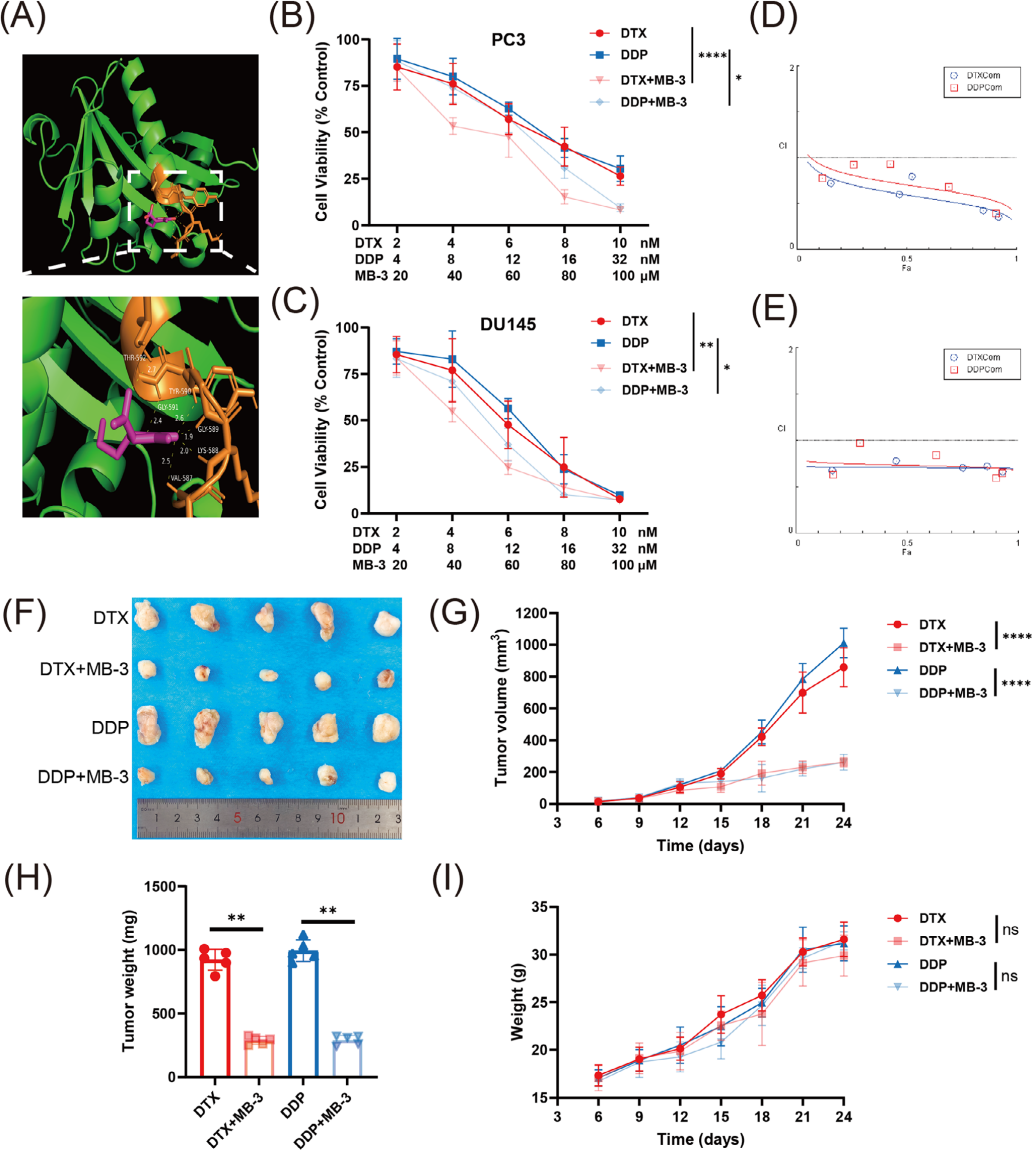
The KAT2A inhibitor Butyrolactone 3 (MB‐3) sensitises prostate cancer cells to docetaxel and cisplatin treatment. (A) Predicted binding pose of MB‐3 within the KAT2A binding pocket from molecular docking analysis (binding energy: –5.5 kcal/mol). (B, C) Cell viability determined by CCK‐8 assays 72 h after treatment with the indicated concentrations of docetaxel, cisplatin, or their combinations with MB‐3 in PC3 (B) and DU145 (C) cells. (D, E) Analysis of drug combination effects (docetaxel or cisplatin with MB‐3) using CompuSyn software, (D) PC3 cells, (E) DU145 cells. A Combination Index (CI) < 1 indicates synergy. (F) Representative images of subcutaneous tumours from nude mice treated with docetaxel or cisplatin alone or in combination with MB‐3; xenografts were generated by subcutaneous inoculation of docetaxel‐resistant or cisplatin‐resistant PC3 cells. (G) Tumour growth curves measured every 3 days. (H) Tumour weight at the experimental endpoint. (I) Body weights monitored every 3 days throughout the treatment period. Data are presented as mean ± SD. Statistical significance was defined as follows: *p < 0.05, **p < 0.01, ***p < 0.001, ****p < 0.0001; ns indicates not significant.

Based on the aforementioned targeting significance, we evaluated the anti‐tumour potential of the reported KAT2A inhibitor MB‐3.^31^ Molecular docking analysis predicted that MB‐3 could stably bind to KAT2A's active site (Figure 8A), supporting its potential to bind directly to the active site of KAT2A. More importantly, CCK‐8 assays demonstrated that MB‐3 combined with docetaxel or cisplatin significantly enhanced chemotherapeutic drug cytotoxicity (Figure 8B and C).

To quantitatively evaluate combination effects, we used CompuSyn software to calculate combination indices (CI).^32^ Results showed that in both cell lines, combination CI values for MB‐3 with docetaxel or cisplatin were all less than 1 (Figure 8D and E), indicating significant synergistic anti‐tumour effects. This in vitro synergy provided the basis for further in vivo validation.

We therefore established xenograft models using docetaxel‐resistant and cisplatin‐resistant PC3 cells to determine whether MB‐3 could improve chemotherapy efficacy in the context of acquired resistance. Compared with either single agent, MB‐3 plus chemotherapy produced substantially stronger tumour suppression, as shown by representative tumour images (Figure 8F), tumour‐growth curves (Figure 8G), and terminal tumour weights (Figure 8H). Body weight did not differ significantly among treatment groups during the experiment (Figure 8I). Together, these data indicate that MB‐3 can resensitise chemoresistant prostate cancer to docetaxel and cisplatin in vivo.

Given that most patients initially respond to chemotherapy, we next evaluated whether MB‐3 could further enhance chemosensitivity in the upfront, chemotherapy‐responsive setting by establishing subcutaneous xenografts using parental PC3 cells. Consistently, MB‐3 combination therapy showed superior antitumour efficacy relative to chemotherapy alone in this chemosensitive model (Figure S6A–D). Importantly, combined MB‐3 treatment did not exacerbate toxic injury in major organs, including the heart, liver, spleen, and lungs (Figure S6E). Together, these results suggest that, under the dosing and administration schedule employed in this study, MB‐3 combined with chemotherapy is well tolerated in the short term and may provide additional therapeutic benefit not only in chemoresistant tumours but also in chemotherapy‐sensitive disease during initial treatment.

## 4 DISCUSSION

This study systematically investigated the role of succinylation in prostate cancer progression and chemotherapy resistance through an integrated framework spanning bioinformatic analyses and functional validation in vitro and in vivo. We developed and validated a novel succinylation score based on transcriptomic data, which effectively stratified patients with prostate cancer into subgroups with distinct prognoses. A high SS was associated with favourable clinical outcomes, including prolonged biochemical recurrence‐free survival, lower Gleason scores, and reduced lymph node metastasis. Mechanistically, a high SS delineated an immunostimulatory TME characterised by enhanced infiltration of cytotoxic immune cells, particularly CD8^+^ precursor exhausted T cells, together with elevated activity of immune activation pathways. Importantly, this immunogenic state was positively associated with enhanced chemotherapy sensitivity, providing an immunological basis for the favourable treatment response observed in high‐SS patients. In contrast, low‐SS tumours displayed an immunosuppressive phenotype accompanied by increased chemoresistance and invasiveness. Further investigation identified KAT2A as a key oncogenic driver in low‐SS tumours. Functional validation confirmed that KAT2A promotes resistance to docetaxel and cisplatin both in vitro and in vivo. Mechanistically, KAT2A catalyses the succinylation of PIK3R2, which competitively inhibits its ubiquitination and subsequent proteasomal degradation, thereby stabilising PIK3R2 and driving chemoresistance. Finally, we demonstrated that the KAT2A inhibitor MB‐3 synergises with standard chemotherapeutic agents to significantly suppress tumour growth, highlighting the therapeutic potential of targeting this pathway to reverse chemoresistance. By presenting results from both chemoresistant and parental models, we further showed that MB‐3 combined with chemotherapy can not only reverse established chemoresistance but also enhance therapeutic efficacy in the initial chemotherapy‐sensitive setting.

A major innovation of this study is the identification of a positive association between a high SS and both favourable prognosis and chemotherapy sensitivity in patients with prostate cancer. This contrasts with findings in some other cancer types, in which higher activity of certain metabolic programs is often associated with enhanced tumour aggressiveness, highlighting the cancer‐type‐specific nature of metabolic regulation.^33^, ^34^ Our data suggest that, in prostate cancer, a relatively intact succinylation network may be associated with a higher degree of tumour differentiation, preservation of specific metabolic functions, and the establishment of a more immunogenic TME, thereby creating favourable conditions for chemotherapeutic agents to exert their effects. Particularly important is the association between high SS and the enrichment of CD8^+^ precursor exhausted T cells (Tpex cells). Tpex cells possess stem‐like features and can be reinvigorated by immunotherapy,^35^, ^36^ and their enrichment may not only enhance immune activity within the TME but also indirectly improve the efficacy of chemotherapy by increasing tumour immunogenicity.^37^ This may help explain the favourable chemotherapy response observed in high‐SS patients, positioning the succinylation score as a biomarker with dual value for prognostic assessment and chemotherapy sensitivity prediction.

Another important finding is the distinct alteration in the expression pattern of succinylation‐related enzymes in tumours. Such patterns have been reported in multiple malignancies, suggesting that they may represent a common event during tumourigenesis.^9^, ^38^ Although these enzymes often exhibit coordinated expression in other tumour types, prostate cancer tissues showed a distinct pattern characterised by downregulation of most enzymes and upregulation of KAT2A. This imbalance may reflect a compensatory response of prostate tumour cells to metabolic stress, although the underlying regulatory mechanisms require further investigation. Notably, depletion of KAT2A had only a minimal effect on the basal proliferative capacity of tumour cells, yet it markedly enhanced sensitivity to chemotherapeutic treatment. This observation suggests that the oncogenic role of KAT2A is context‐dependent, primarily mediating the adaptive response of tumour cells to chemotherapy rather than regulating baseline growth. These findings challenge the potential misconception that KAT2A directly drives tumour proliferation and provide a new direction for the rational design of strategies aimed at reversing chemoresistance.^39^, ^40^

A key conceptual point to clarify is the apparent contrast between the favourable prognosis of high‐SS tumours and the oncogenic role of KAT2A, a succinyltransferase that is upregulated in low‐SS chemoresistant tumours. This apparent paradox is resolved by distinguishing between two distinct biological levels. First, the succinylation score (SS) reflects a global transcriptomic state of the overall succinylation–metabolic network, correlating with tumour differentiation status and the immunostimulatory tumour microenvironment (TME). High SS thus indicates a relatively intact metabolic program and a more immunogenic tumour ecology, which together correspond to better prognosis. Second, KAT2A upregulation represents a compensatory, substrate‐specific event that occurs specifically in the context of global succinylation deficiency (low SS). Under these conditions, tumour cells hijack KAT2A to mediate site‐specific succinylation of a limited set of non‐histone substrates—such as PIK3R2. This localised modification does not restore global metabolic homeostasis, but rather selectively stabilises key survival signalling nodes, thereby driving chemoresistance. In essence, SS marks the overall tumour differentiation and immune contexture, whereas KAT2A functions as a specific adaptive effector that mediates resistance when canonical metabolic programs are disrupted. Operating at different biological levels, these two phenomena are not mutually contradictory.

Furthermore, to our knowledge, this study is the first to reveal a molecular mechanism by which KAT2A promotes chemoresistance through stabilisation of PIK3R2 and to clarify the association of this process with the immune microenvironment. Although KAT2A is well known as a histone acetyltransferase,^41^, ^42^ we additionally identified its role as a succinyltransferase in regulating the stability of the non‐histone protein PIK3R2, thereby expanding the scope of its oncogenic functions. Importantly, we evaluated whether the classical acetyltransferase activity of KAT2A contributes to the observed phenotypes. Our experimental data showed that neither KAT2A knockout nor overexpression significantly altered the acetylation level of PIK3R2 in prostate cancer cell lines or human organoid models. In addition, bioinformatic analysis revealed that the predicted acetylation residues of PIK3R2 are distinct from and do not overlap with the identified succinylation sites (K477 and K564), suggesting that these two post‐translational modifications are regulated independently. These findings support the conclusion that the observed chemoresistant phenotypes are primarily attributable to KAT2A‐mediated succinylation rather than its classical acetyltransferase function. Although our site‐directed mutagenesis data identify K477 and K564 as key residues involved in KAT2A‐dependent succinylation and support functional competition with ubiquitination at these lysine sites, the precise residue‐level structural dynamics of this competition require further investigation. Future studies incorporating more detailed biochemical and structural analyses, as well as downstream pathway readouts such as PI3K/AKT activation, DNA damage signalling, and apoptosis markers, will help further refine this mechanism.

Despite these advances, this study has several limitations. The succinylation score was developed and validated using retrospective data from The Cancer Genome Atlas, and its clinical applicability and generalisability therefore require further verification in larger, multicentre prospective studies. Prospective clinical studies will be needed to validate the value of SS as a companion diagnostic tool and to systematically evaluate its correlation and complementarity with existing clinical biomarkers, such as PSA and Gleason score. Although single‐cell RNA sequencing provided high‐resolution insights into the TME, the limited sample size necessitates confirmation in independent cohorts. While pharmacological data support the targetability of KAT2A, the efficacy and safety of KAT2A inhibitors (including MB‐3) require rigorous preclinical and clinical evaluation. Similarly, organoid‐based validation was performed in only four patient‐derived models and should be regarded as proof‐of‐concept rather than definitive clinical stratification. Larger organoid collections or patient‐derived xenograft cohorts will be needed to further validate the translational relevance of KAT2A‐associated chemosensitivity. To address these issues, future work will focus on three directions: establishing patient‐derived xenograft biobanks with greater molecular diversity to assess the predictive power and therapeutic utility of SS; exploring optimal combination regimens of KAT2A inhibitors with chemotherapy; and developing immunotherapy‐based combination strategies centred on succinylation modulation.

From a translational perspective, MB‐3 should currently be regarded as a tool compound that pharmacologically supports the targetability of KAT2A, rather than as a clinically optimised therapeutic candidate. Although our in vivo studies showed that MB‐3 enhanced chemotherapy efficacy and did not cause obvious histopathological abnormalities in major organs, including the heart, liver, spleen, lungs, and kidneys, under the dosing conditions used in this study, its selectivity, off‐target effects, pharmacokinetic properties, bioavailability, and long‐term safety remain to be systematically characterised. Therefore, additional medicinal chemistry optimisation and preclinical pharmacology studies will be required before KAT2A‐targeted strategies can be translated into clinical application.

In summary, this study establishes the succinylation score as a robust biomarker integrating metabolic and immune features in prostate cancer. It identifies the KAT2A–PIK3R2 succinylation axis as a novel mechanism driving chemoresistance, thereby challenging the conventional view that simplistically links metabolic activation to tumour promotion. Our findings highlight the context‐dependent role of specific post‐translational modifications in prostate cancer progression. The proposed model suggests that succinylation levels influence tumour microenvironment composition, immune cell function, and chemotherapy sensitivity, providing a new conceptual framework for understanding tumour heterogeneity. Clinically, the succinylation score has potential to improve risk stratification and personalise treatment by identifying chemotherapy‐sensitive patients. Moreover, the combination of the KAT2A inhibitor MB‐3 with chemotherapy represents a promising strategy for reversing chemoresistance with clear translational potential. Overall, this work connects metabolic regulation, immune contexture, and therapy response, and thereby advances precision oncology in advanced prostate cancer.

## 5 CONCLUSION

This study establishes the succinylation score as a novel prognostic biomarker for prostate cancer, with high scores associated with an immunostimulatory tumour microenvironment and increased chemotherapy sensitivity. We identified KAT2A as a key driver of chemoresistance and demonstrated that it stabilises PIK3R2 through succinylation, thereby promoting drug resistance. Importantly, the KAT2A inhibitor MB‐3 showed synergistic effects with standard chemotherapy in suppressing tumour growth. Collectively, these findings provide both a new stratification tool and a promising therapeutic strategy for overcoming chemoresistance in advanced prostate cancer.

## AUTHOR CONTRIBUTIONS

A.D., W.H., and Z.L. conducted the experiments, analysed the data, and co‐wrote the manuscript. L.L. and K.L. provided technical support and assisted with data collection. S.F. and D.X. conceived and supervised the project, acquired funding, and finalised the manuscript. All authors reviewed and approved the final version.

## CONFLICT OF INTEREST STATEMENT

The authors have stated that there are no conflicts of interest.

## ETHICS STATEMENT

The study involving human participants was reviewed and approved by the Medical Research Ethics Committee of The Second Hospital of Anhui Medical University (Approval No. SL‐YX2023‐167). All participants provided written informed consent prior to enrolment. The animal experiments were conducted in accordance with institutional guidelines and were approved by the Animal Welfare and Ethics Committee of the Institute of Health and Medicine, Hefei Comprehensive National Science Center (Approval No. IHM‐AP‐2024‐046). All procedures followed the relevant national and institutional guidelines for the care and use of laboratory animals.

## Supporting information



SUPPORTING INFORMATION

SUPPORTING INFORMATION

## Data Availability

All data supporting the findings of this study are available within the article and its supplementary materials. Publicly available datasets used in this study were obtained from The Cancer Genome Atlas (TCGA) Prostate Adenocarcinoma (PRAD) cohort via the UCSC Xena browser and from the Gene Expression Omnibus (GEO) under accession number GSE137829. Additional data generated during this study are available from the corresponding authors upon reasonable request.

## References

[1] Kratzer TB , Mazzitelli N , Star J , Dahut WL , Jemal A , Siegel RL . Prostate Cancer Statistics, 2025. CA Cancer J Clin. 2025;75(6):485‐497. 40892160 10.3322/caac.70028 PMC12593258

[2] Lycken M , Bergengren O , Drevin L , et al. Changes in characteristics of men with lethal prostate cancer during the past 25 years: description of population‐based deaths. Eur Urol Open Sci. 2022;41:81‐87. 35813253 10.1016/j.euros.2022.05.003 PMC9257655

[3] Min Y , Wei X , Chen H , Xiang K , Yin G , Feng Y . Identifying clinicopathological risk factors of the regional lymph node metastasis in patients with T(1‐2) mucinous breast cancer: a population‐based study. J Oncol. 2021;2021:3866907. 34306075 10.1155/2021/3866907 PMC8285172

[4] Lu J , Zou Q , Li Y , et al. Fth1p8 induces and transmits docetaxel resistance by inhibiting ferroptosis in prostate cancer. Biomed Pharmacother. 2024;180:117472. 39332191 10.1016/j.biopha.2024.117472

[5] Wu T , Zhao Y , Zhang X , et al. Short‐chain acyl post‐translational modifications in cancers: mechanisms, roles, and therapeutic implications. Cancer Commun (Lond). 2025;45(10):1247‐1284. 40703012 10.1002/cac2.70048 PMC12531430

[6] He S , Wang C , Li R , et al. The role of succinylation‐mediated metabolic reprogramming in tumor progression. Mol Biol Rep. 2025;52(1):954. 41003805 10.1007/s11033-025-11061-6

[7] Lu K , Han D . A review of the mechanism of succinylation in cancer. Medicine (Baltimore). 2022;101(45):e31493. 36397343 10.1097/MD.0000000000031493 PMC9666091

[8] Rardin MJ , He W , Nishida Y , et al. Sirt5 regulates the mitochondrial lysine succinylome and metabolic networks. Cell Metab. 2013;18(6):920‐933. 24315375 10.1016/j.cmet.2013.11.013 PMC4105152

[9] Shen R , Ruan H , Lin S , et al. Lysine succinylation, the metabolic bridge between cancer and immunity. Genes Dis. 2023;10(6):2470‐2478. 37554179 10.1016/j.gendis.2022.10.028 PMC10404875

[10] Zhang N , Sun L , Zhou S , et al. Cholangiocarcinoma Pdha1 succinylation suppresses macrophage antigen presentation via alpha‐ketoglutaric acid accumulation. Nature Communications. 2025;16(1):3177. 10.1038/s41467-025-58429-7 PMC11968997 40180922

[11] C H Chang , Qiu J , O'Sullivan D , et al. Metabolic competition in the tumor microenvironment is a driver of cancer progression. Cell. 2015;162(6):1229‐1241. 26321679 10.1016/j.cell.2015.08.016 PMC4864363

[12] Zou W , Green DR . Beggars Banquet: metabolism in the tumor immune microenvironment and cancer therapy. Cell Metab. 2023;35(7):1101‐1113. 37390822 10.1016/j.cmet.2023.06.003 PMC10527949

[13] Chen X , Chen S , Yu D . metabolic reprogramming of chemoresistant cancer cells and the potential significance of metabolic regulation in the reversal of cancer chemoresistance. Metabolites. 2020;10(7):289. 32708822 10.3390/metabo10070289 PMC7408410

[14] Ren X , Wang X , Zheng G , et al. Targeting one‐carbon metabolism for cancer immunotherapy. Clin Transl Med. 2024;14(1):e1521. 38279895 10.1002/ctm2.1521 PMC10819114

[15] Kwon OK , Bang IH , Choi SY , et al. Ldha desuccinylase Sirtuin 5 as a novel cancer metastatic stimulator in aggressive prostate cancer. Genomics Proteomics Bioinformatics. 2023;21(1):177‐189. 35278714 10.1016/j.gpb.2022.02.004 PMC10372916

[16] Pujana‐Vaquerizo M , Bozal‐Basterra L , Carracedo A . Metabolic adaptations in prostate cancer. Br J Cancer. 2024;131(8):1250‐1262. 38969865 10.1038/s41416-024-02762-z PMC11473656

[17] Zhang W , Meng Y , Liu N , Wen XF , Yang T . Insights into chemoresistance of prostate cancer. Int J Biol Sci. 2015;11(10):1160‐1170. 26327810 10.7150/ijbs.11439 PMC4551752

[18] Dong B , Miao J , Wang Y , et al. Single‐cell analysis supports a luminal‐neuroendocrine transdifferentiation in human prostate cancer. Commun Biol. 2020;3(1):778. 33328604 10.1038/s42003-020-01476-1 PMC7745034

[19] Butler A , Hoffman P , Smibert P , Papalexi E , Satija R . Integrating Single‐cell transcriptomic data across different conditions, technologies, and species. Nat Biotechnol. 2018;36(5):411‐420. 29608179 10.1038/nbt.4096 PMC6700744

[20] Stuart T , Butler A , Hoffman P , et al. Comprehensive integration of single‐cell data. Cell. 2019;177(7):1888‐1902 e21. 10.1016/j.cell.2019.05.031 PMC6687398 31178118

[21] Korsunsky I , Millard N , Fan J , et al. Fast, sensitive and accurate integration of single‐cell data with harmony. Nat Methods. 2019;16(12):1289‐1296. 31740819 10.1038/s41592-019-0619-0 PMC6884693

[22] Ritchie ME , Phipson B , Wu D , et al. limma powers differential expression analyses for RNA‐sequencing and microarray studies. Nucleic Acids Res. 2015;43(7):e47. 25605792 10.1093/nar/gkv007 PMC4402510

[23] Jiang P , Gu S , Pan D , et al. Signatures of T cell dysfunction and exclusion predict cancer immunotherapy response. Nat Med. 2018;24(10):1550‐1558. 30127393 10.1038/s41591-018-0136-1 PMC6487502

[24] Maeser D , Gruener RF , Huang RS . Oncopredict: an R package for predicting in vivo or cancer patient drug response and biomarkers from cell line screening data. Brief Bioinform. 2021;22(6):bbab260. 34260682 10.1093/bib/bbab260 PMC8574972

[25] Wang P , Henning SM , Magyar CE , Elshimali Y , Heber D , Vadgama JV . Green tea and quercetin sensitize PC‐3 xenograft prostate tumors to docetaxel chemotherapy. J Exp Clin Cancer Res. 2016;35:73. 27151407 10.1186/s13046-016-0351-x PMC4858851

[26] Festuccia C , Gravina GL , D'Alessandro AM , et al. Azacitidine improves antitumor effects of docetaxel and cisplatin in aggressive prostate cancer models. Endocr Relat Cancer. 2009;16(2):401‐413. 19153211 10.1677/ERC-08-0130

[27] Zhang Y , Gao Y , Ding Y , et al. Targeting Kat2a inhibits inflammatory macrophage activation and rheumatoid arthritis through epigenetic and metabolic reprogramming. MedComm (2020). 2023;4(3):e306. 37313329 10.1002/mco2.306 PMC10258526

[28] Zhang Y , Zhang H , Wang H , et al. Tandem mass tag‐based quantitative proteomic analysis identification of succinylation related proteins in pathogenesis of thoracic aortic aneurysm and aortic dissection. PeerJ. 2023;11:e15258. 37193023 10.7717/peerj.15258 PMC10183161

[29] Zhang H , Ling M , Zhang Y , Fang Q , Wo W , Lv X . Oxct1 promotes triple negative breast cancer immune escape via modulating succinylation modification of Pgk1. Commun Biol. 2025;8(1):1033. 40634657 10.1038/s42003-025-08433-w PMC12241480

[30] He M , Yang Z , Xie L , et al. Rnf167 mediates atypical ubiquitylation and degradation of Rlrs via two distinct proteolytic pathways. Nature Communications. 2025;16(1):1920. 10.1038/s41467-025-57245-3 PMC11850712 39994288

[31] Li J , Yan C , Wang Y , et al. Gcn5‐mediated regulation of pathological cardiac hypertrophy via activation of the Tak1‐Jnk/P38 signaling pathway. Cell Death & Disease. 2022;13(4):421. 35490166 10.1038/s41419-022-04881-y PMC9056507

[32] Du L , Liu W , Pichiorri F , Rosen ST . Sumoylation inhibition enhances multiple myeloma sensitivity to lenalidomide. Cancer Gene Ther. 2023;30(4):567‐574. 35338347 10.1038/s41417-022-00450-9 PMC10104776

[33] Ou B , Liu Y , Yang X , Xu X , Yan Y , Zhang J . C5aR1‐positive neutrophils promote breast cancer glycolysis through WTAP‐dependent m6A methylation of ENO1. cell death & disease. 2021;12(8):737. 34312368 10.1038/s41419-021-04028-5 PMC8313695

[34] Yan J , Chen D , Ye Z , et al. Molecular mechanisms and therapeutic significance of tryptophan metabolism and signaling in cancer. Mol Cancer. 2024;23(1):241. 39472902 10.1186/s12943-024-02164-y PMC11523861

[35] Neubert EN , DeRogatis JM , Lewis SA , et al. Hmgb2 regulates the differentiation and stemness of Exhausted Cd8(+) T cells during chronic viral infection and cancer. Nature Communications. 2023;14(1):5631. 10.1038/s41467-023-41352-0 PMC10499904 37704621

[36] Zou D , Li XC , Chen W . Beyond T‐cell subsets: stemness and adaptation redefining immunity and immunotherapy. Cell Mol Immunol. 2025;22(9):957‐974. 40634636 10.1038/s41423-025-01321-7 PMC12398556

[37] Liu S , Li J , Gu L , Wu K , Xing H . Nanoparticles for chemoimmunotherapy against triple‐negative breast cancer. Int J Nanomedicine. 2022;17:5209‐5227. 36388877 10.2147/IJN.S388075 PMC9651025

[38] Wang Y , Guo YR , Liu K , et al. Kat2a coupled with the Alpha‐Kgdh complex acts as a histone H3 succinyltransferase. Nature. 2017;552(7684):273‐277. 29211711 10.1038/nature25003 PMC5841452

[39] Liu K , Zhang Q , Lan H , et al. Gcn5 potentiates glioma proliferation and invasion via Stat3 and Akt signaling pathways. Int J Mol Sci. 2015;16(9):21897‐21910. 26378521 10.3390/ijms160921897 PMC4613287

[40] Tong Y , Guo D , Yan D , et al. Kat2a succinyltransferase activity‐mediated 14‐3‐3zeta upregulation promotes beta‐catenin stabilization‐dependent glycolysis and proliferation of pancreatic carcinoma cells. Cancer Lett. 2020;469:1‐10. 31610265 10.1016/j.canlet.2019.09.015

[41] Li H , Li C , Yang LZ , Liu J . Integrative analysis of histone acetyltransferase Kat2a in human cancer. Cancer Biomark. 2023;38(4):443‐463. 38007639 10.3233/CBM-220464 PMC12412876

[42] White J , Derheimer FA , Jensen‐Pergakes K , et al. Histone lysine acetyltransferase inhibitors: an emerging class of drugs for cancer therapy. Trends Pharmacol Sci. 2024;45(3):243‐254. 38383216 10.1016/j.tips.2024.01.010

