# Supplementary material for: A succinylation‐based classifier predicts chemotherapy response in prostate cancer and reveals KAT2A as a therapeutic target
Source: Clin Transl Med. 2026 Jul 7;16(7):e70737. doi: 10.1002/ctm2.70737 (PMC13341643; doi:10.1002/ctm2.70737)
Supplement: Supplementary file 1 — SUPPORTING INFORMATION [file CTM2-16-e70737-s002.docx]

**Supplementary Table 1. Plasmid information used in current work.**

| **Gene_ID** | **Vector** | **Application** | **Sequence** |
| --- | --- | --- | --- |
| KAT2A^#1^ | PX459 | Knockout | F: CCTGGAGCGCAAGATCAACG |
|  |  |  | R: GTTGATCTTGCGCTCCAGC |
| KAT2A^#2^ | PX459 | Knockout | F: GCGCTACTTCTCCGACG |
|  |  |  | R: CGTCGGAGAAGTAGCGCCC |
| KAT2A^#3^ | PX459 | Knockout | F: AGTGGGACGCGCTGCACGA |
|  |  |  | R: TCGTGCAGCGCGTCCCACTC |
| KAT2A | pCMV | Overexpression | F: GCCACCATGGCCGCCGCCGCCGCC |
|  |  |  | R: CTTGTCGTCATCGTCTTTGTAGTCCTACGCGTCCTCCTTGGC |

**Supplementary Table 2. The primers sequences used in current work.**

| **Primer name** | **Sequence** |
| --- | --- |
| KAT2A  BBC3 | F: GGACATCAAGGTGCCCAAGA  R: TCAGCTTCTTGATGATCACCTC  F: GAGCAGGGCAGGAAGTAACA |
|  | R: CTCCCTGGGGCCACAAATC |
| PIK3R2 | F: GAGACCAGTACCTCGTGTGG |
|  | R: ATCGTCCTCGTCCTCCATGA |
| GAPDH | F: GGAGCGAGATCCCTCCAAAAT  R: GGCTGTTGTCATACTTCTCATGG |
| ACTIN | F: CTGTGGCATCCACGAAACTA  R: AGTACTTGCGCTCAGGAGGA |
