# Supplementary material for: A succinylation‐based classifier predicts chemotherapy response in prostate cancer and reveals KAT2A as a therapeutic target
Source: Clin Transl Med. 2026 Jul 7;16(7):e70737. doi: 10.1002/ctm2.70737 (PMC13341643; doi:10.1002/ctm2.70737)
Supplement: Supplementary file 2 — SUPPORTING INFORMATION [file CTM2-16-e70737-s001.docx]

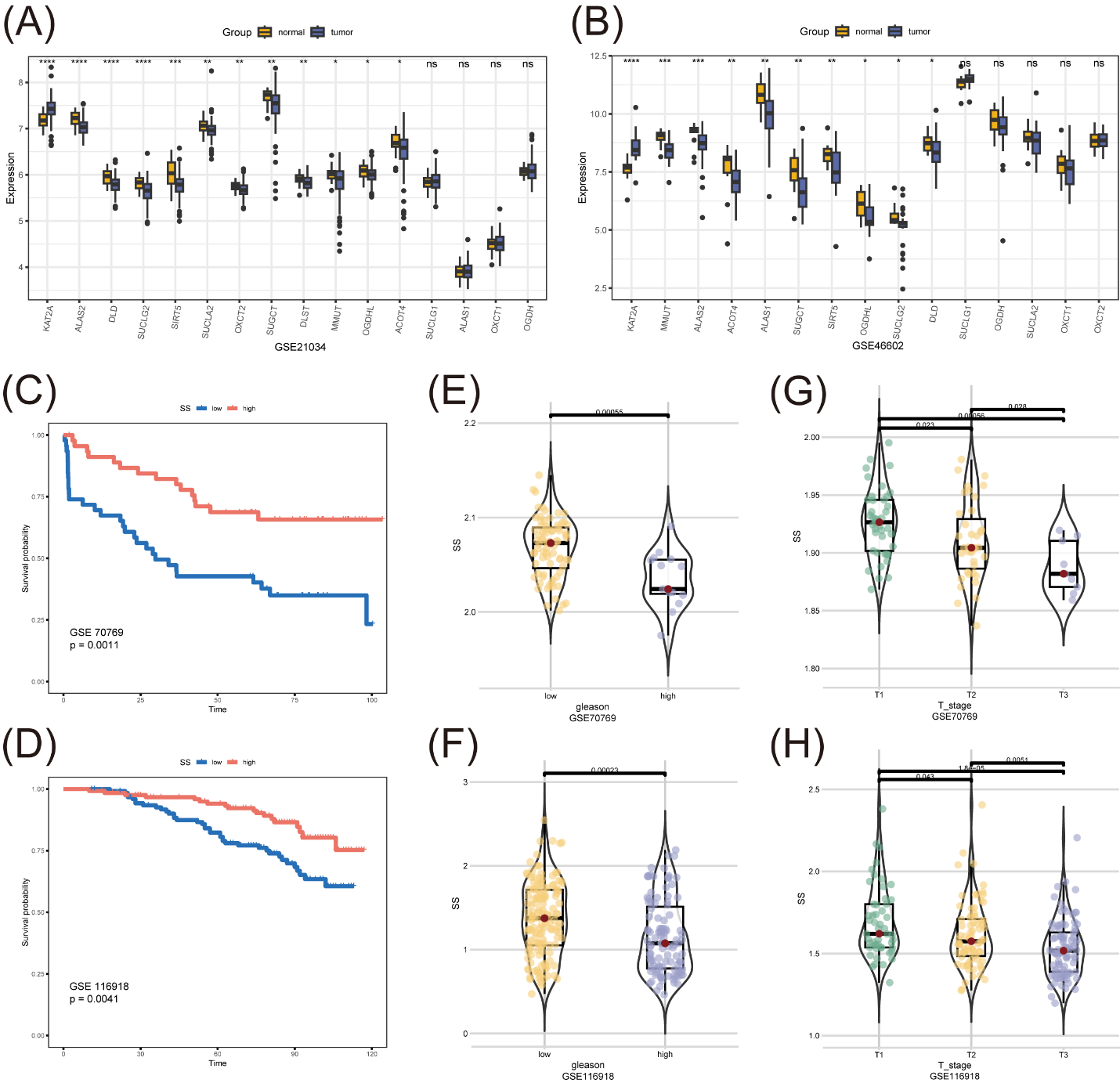


Figure S1. Validation of succinylation-related gene expression and clinical associations in independent prostate cancer cohorts.
(A, B) Boxplots comparing the expression levels of succinylation-related genes between tumor and adjacent normal tissues in the GSE21034 (A) and GSE46602 (B) cohorts. KAT2A is consistently upregulated in tumors, whereas most other succinylation-related enzymes (SUCLA2, SUCLG1, OGDH, OXCT2, DLST, DLD, SIRT5, ACOT4, ALAS1, OXCT1, SUCLG2, OGDHL, SUGCT, ALAS2, MMUT) show downregulation or no significant change, mirroring the pattern observed in the TCGA-PRAD cohort. (C, D) Kaplan–Meier curves of biochemical recurrence-free survival stratified by high‑SS and low‑SS groups (median cutoff) in the GSE70769 (C) and GSE116918 (D) cohorts. Patients with high SS exhibited significantly better prognosis in both datasets (P values by log‑rank test are indicated). (E, F) Distribution of SS according to primary Gleason grade (≤7 vs ≥8) in GSE70769 (E) and GSE116918 (F). High SS is significantly associated with lower Gleason grade. (G, H) Distribution of SS according to tumor stage (T1–T2 vs T3) in GSE70769 (G) and GSE116918 (H). High SS correlates with earlier tumor stage. Data in boxplots are presented as median with interquartile range; whiskers extend to 1.5× IQR. Statistical comparisons were performed using two‑sided Wilcoxon rank‑sum test.


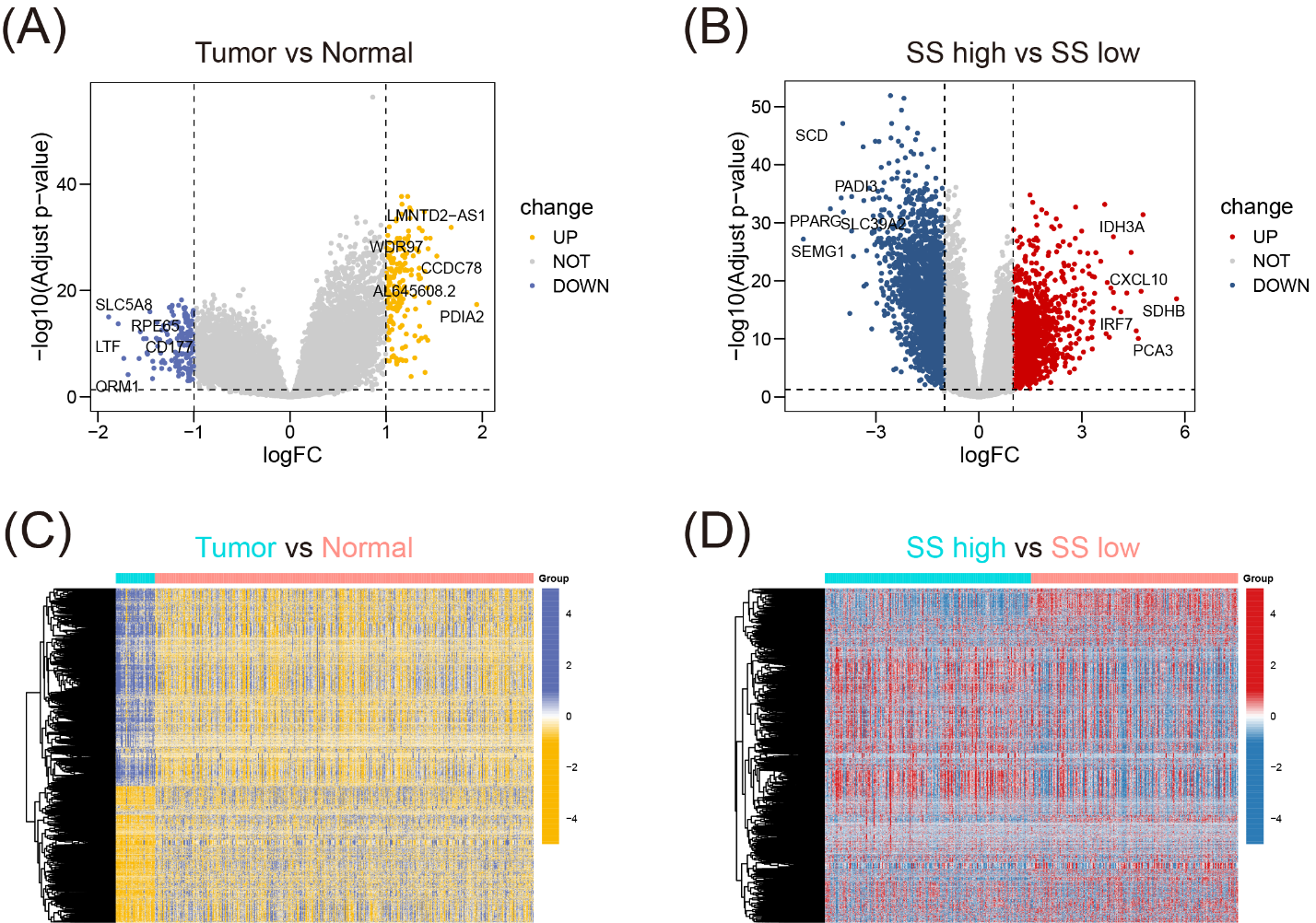


Figure S2. Differential gene expression between tumor vs. normal and high-SS vs. low-SS. (A) Volcano plot of differentially expressed genes (DEGs) between tumor and normal tissues. (B) Volcano plot of DEGs between high-SS and low-SS groups. (C–D) Heatmaps of unsupervised hierarchical clustering based on DEGs between tumor and normal (C), and between high-SS and low-SS groups (D).


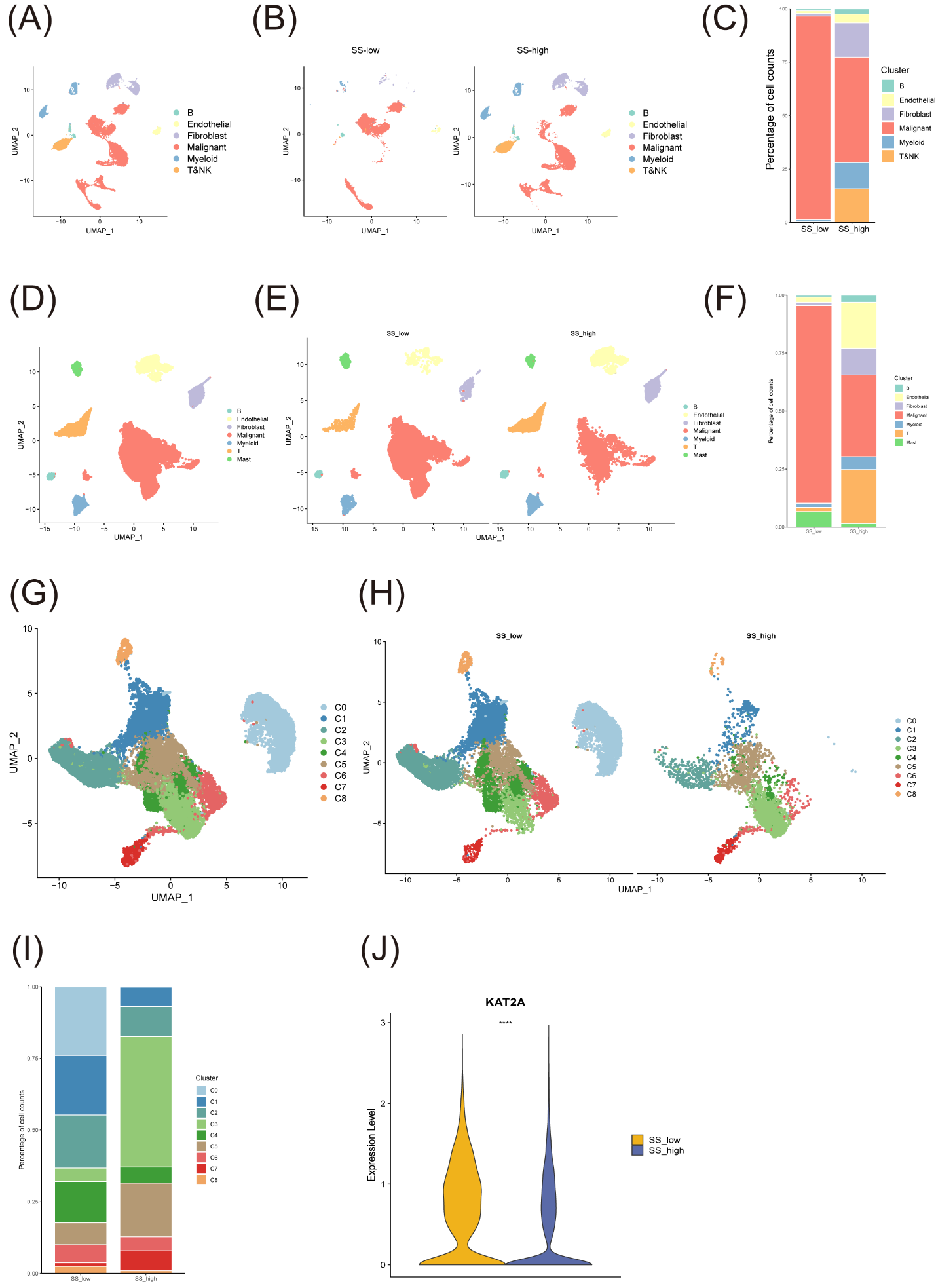


Figure S3. Single-cell TME composition in high-SS vs. low-SS groups. (A) UMAP plot of all cells colored by major cell type annotations. (B) UMAP plots of cells stratified by high-SS and low-SS classification. (C) Barplot showing the percentage of each cell type in high-SS and low-SS groups. (D) UMAP plot of all cells colored by major cell type annotations in the GSE141445 cohort. (E) UMAP plots of cells stratified by high-SS and low-SS classification. (F) Barplot showing the percentage of each cell type in high-SS and low-SS groups. (G) UMAP plot of malignant epithelial cells reclustered into transcriptionally distinct subpopulations. (H) UMAP plots of malignant cells stratified by high-SS and low-SS groups. (I) Barplot showing the percentage distribution of each malignant subcluster in high-SS and low-SS groups. (J) Boxplot comparing KAT2A expression levels in malignant cells between low-SS and high-SS groups.


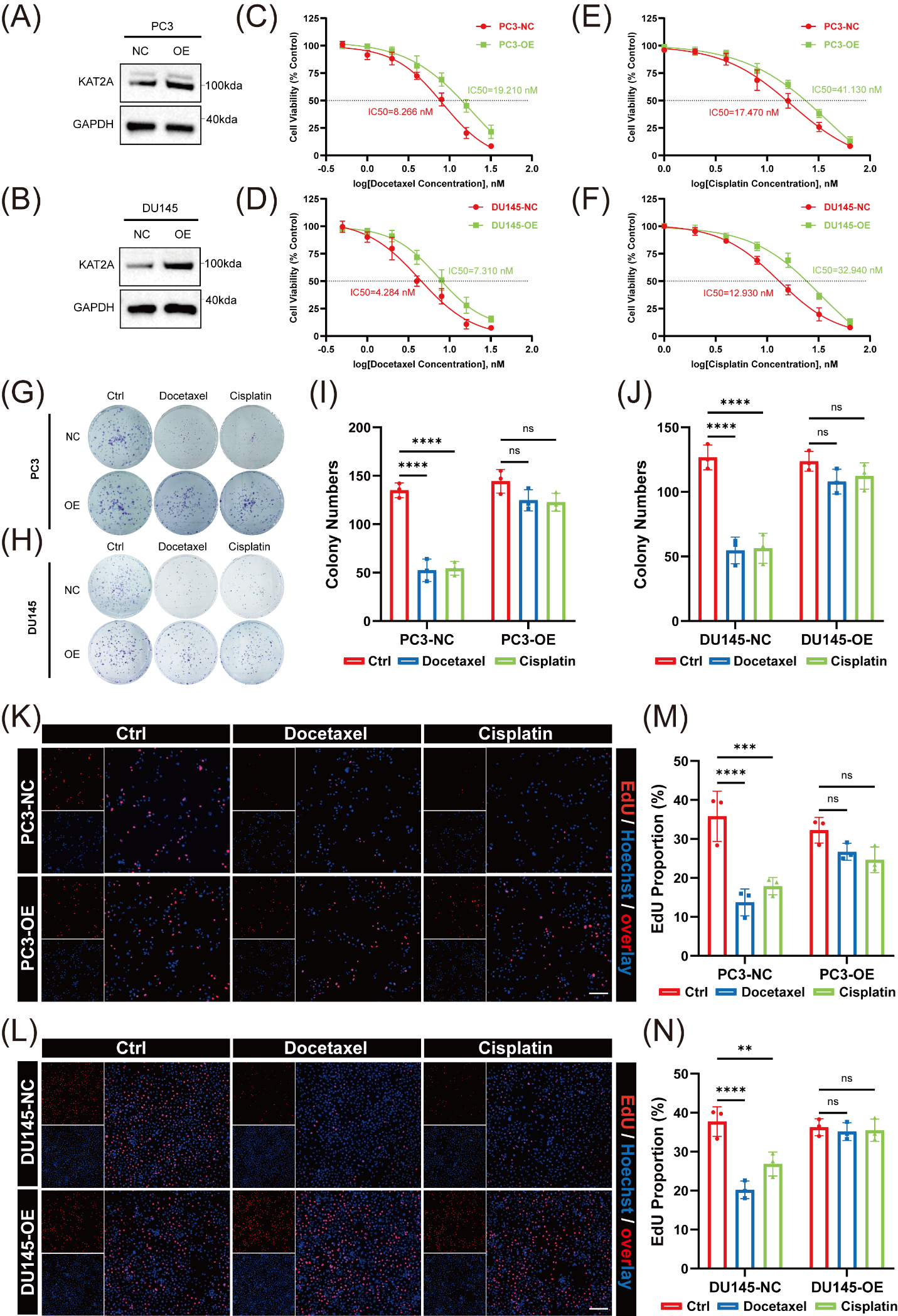


Figure S4. Overexpression of KAT2A reduces the sensitivity of prostate cancer cells to docetaxel and cisplatin treatment in vitro. (A, B) Western blot validation of KAT2A overexpression efficiency in PC3 (A) and DU145 (B) cells. (C, D) Determination of the IC_50_ for docetaxel in PC3 (C) and DU145 (D) cells following KAT2A overexpression, measured using the CCK‑8 assay. (E, F) Determination of IC_50_ values for cisplatin in PC3 (E) and DU145 (F) cells following KAT2A overexpression, measured using the CCK‑8 assay. (G, H) Representative images of colony formation assays for PC3 (G) and DU145 (H) cells after KAT2A overexpression, in the presence of cisplatin or docetaxel. (I, J) Quantification of colonies formed by PC3 (I) and DU145 (J) cells. (K, L) Representative EdU incorporation assay images for PC3 (K) and DU145 (L) cells after KAT2A overexpression, in the presence of cisplatin or docetaxel. Scale bar: 100 µm. (M, N) Quantification of EdU‑positive cells in PC3 (M) and DU145 (N) populations. Data are presented as mean  ±  SD. Statistical significance: **P*  <  0.05; ***P*  <  0.01; ****P*  <  0.001; *****P*<  0.0001.


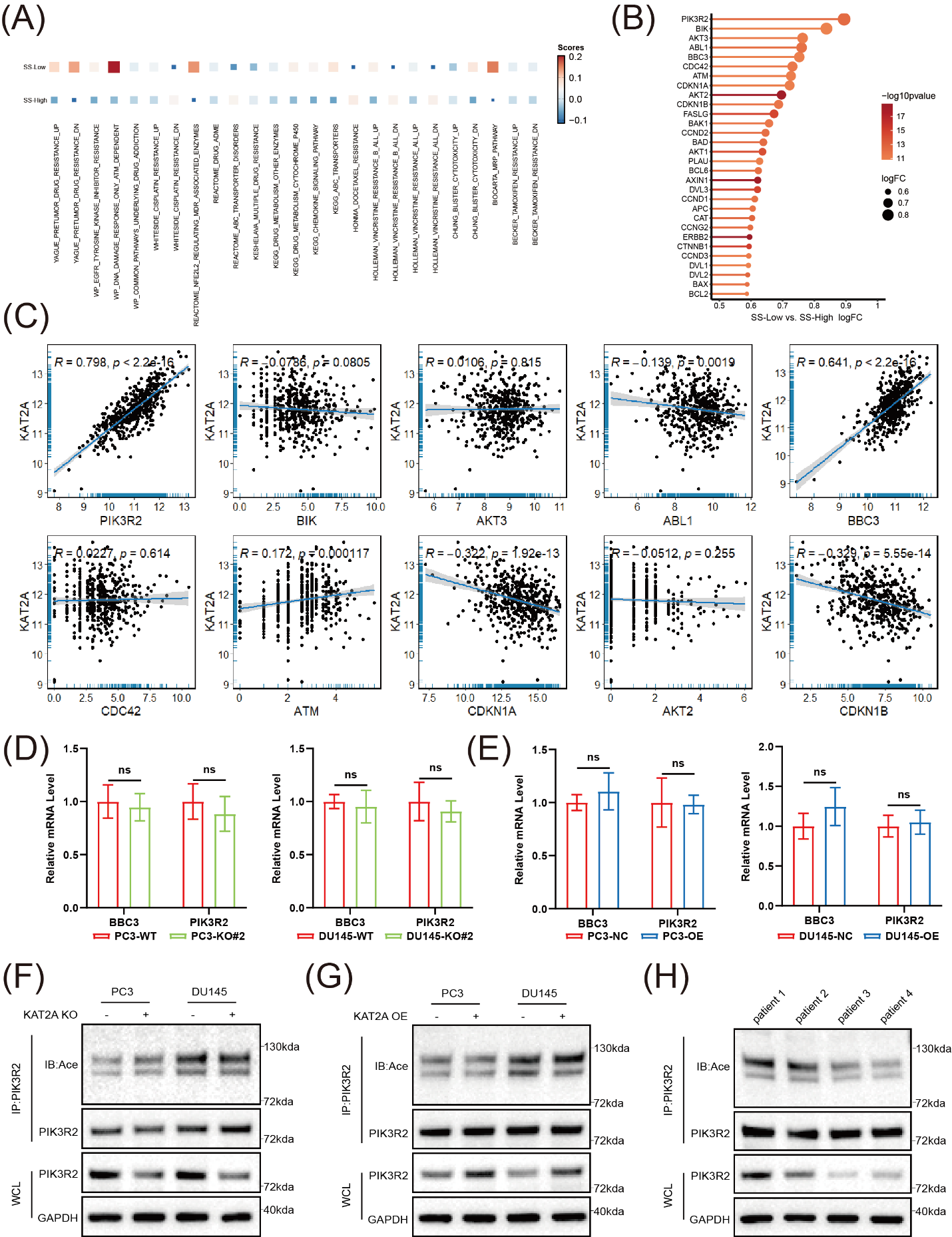


Figure S5. KAT2A Promotes Chemoresistance in Prostate Cancer by Regulating PIK3R2 Succinylation. (A) Pathway enrichment analysis using the MSigDB database reveals significant enrichment of DNA damage response-related pathways in prostate cancer samples from the low-SS group. (B) Among the significantly enriched DNA damage response pathways, the top 30 core genes that are upregulated in the low-SS group are presented. (C) Correlation analysis between KAT2A expression and core genes associated with chemotherapy resistance in prostate cancer. (D, E) Quantitative real-time PCR analysis of PIK3R2 and BBC3 mRNA levels following KAT2A knockout (D) or overexpression (E). (F) Immunoprecipitation (IP) of PIK3R2 followed by immunoblotting (IB) with an anti-acetyl-lysine antibody to assess PIK3R2 acetylation levels after KAT2A knockout. (G) IP of PIK3R2 followed by IB with an anti-acetyl-lysine antibody to assess PIK3R2 acetylation levels after KAT2A overexpression. (H) Assessment of PIK3R2 acetylation in human prostate cancer organoids by IP-IB analysis (IP with anti-PIK3R2; IB with anti-acetyl-lysine).


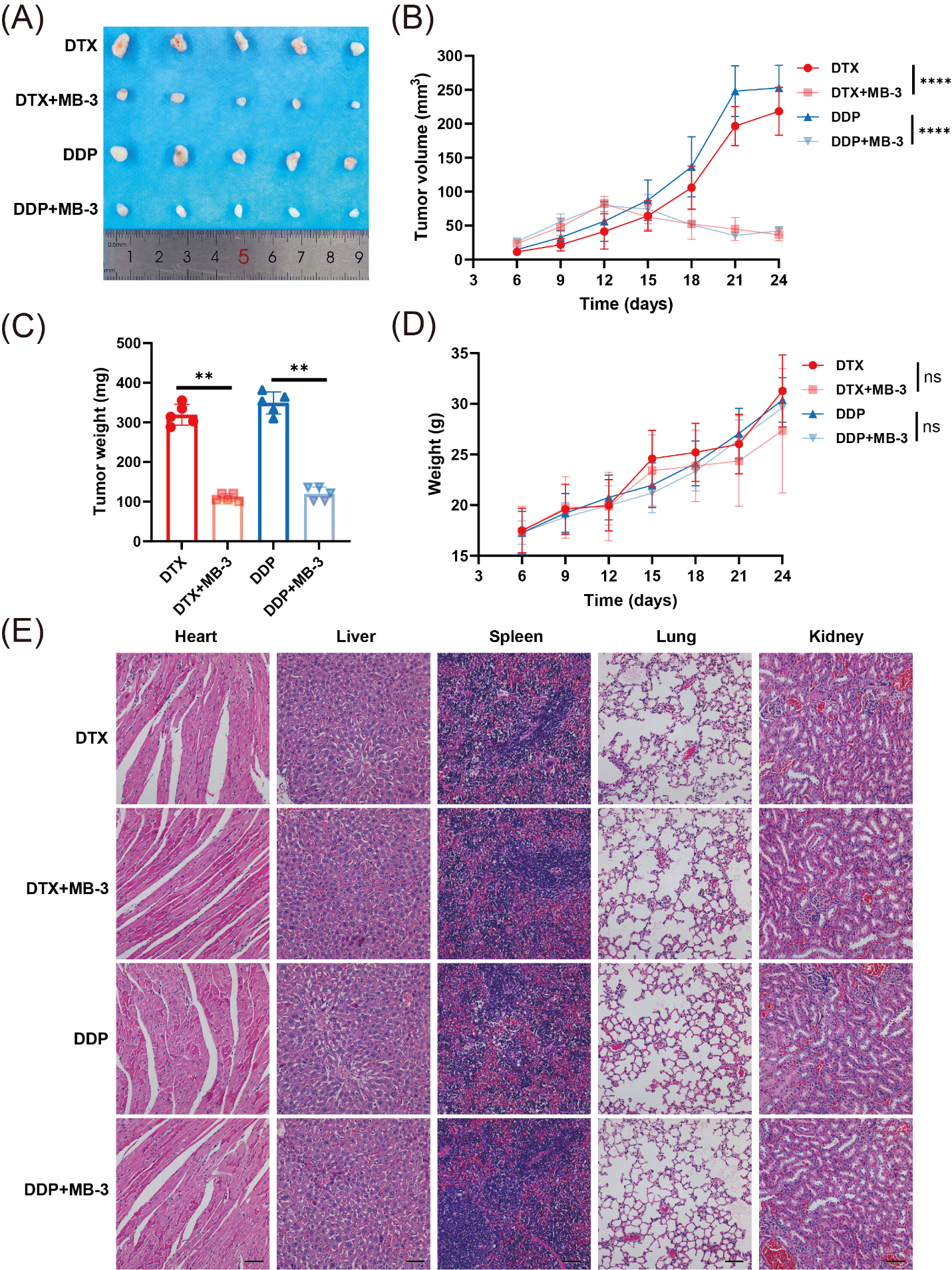


Figure S6. The KAT2A inhibitor Butyrolactone 3 (MB-3) sensitizes prostate cancer cells to docetaxel and cisplatin treatment. (A) Representative images of subcutaneous tumors from nude mice treated with docetaxel or cisplatin alone or in combination with MB-3; xenografts were generated by subcutaneous inoculation of parental PC3 cells. (B) Tumor growth curves measured every three days. (C) Tumor weight at the experimental endpoint. (D) Body weights monitored every 3 days throughout the treatment period. (E) Hematoxylin and eosin (H&E) staining of major organs (heart, liver, spleen, lungs, and kidneys) to evaluate systemic toxicity associated with MB-3 combination therapy. Data are presented as mean ± SD. Statistical significance was defined as follows: **P* < 0.05, ***P* < 0.01, ****P* < 0.001, *****P* < 0.0001; ns indicates not significant.
